# Hydrogeophysical and aquifer vulnerability zonation of a typical basement complex terrain: A case study of Odode Idanre southwestern Nigeria

**DOI:** 10.1016/j.heliyon.2020.e04549

**Published:** 2020-08-18

**Authors:** O.J. Akintorinwa, M.O. Atitebi, A.A. Akinlalu

**Affiliations:** Department of Applied Geophysics, Federal University of Technology, Akure, Nigeria

**Keywords:** Geology, Geophysics, Earth sciences, Hydrology, Environmental science, Aquifer vulnerability, Anisotropy, Hydraulic conductivity, Longitudinal conductance, Multi-criteria technique

## Abstract

An approach engaging Vertical Electrical Sounding (VES) and remote sensing data was carried out with a view to developing groundwater potential and aquifer vulnerability maps of the study area. One hundred and one (101) depth sounding data were acquired using Schlumberger array, with half maximum current electrode separation (AB/2) of 100 m. The VES were quantitatively interpreted using partial curve matching and computer aided iteration to determine the geoelectrical parameters of each station. The remote sensing data were processed using the application of Geographic Information System-based multi-criteria technique ArcGIS software. Eight (8) parameters namely lineament density, drainage density, slope, transmissivity, hydraulic conductivity, coefficient of anisotropy, aquifer thickness and resistivity were used to produce the groundwater potential model while five (5) parameters namely, lineament density, slope, longitudinal conductance, hydraulic conductivity and thickness of layer overlying the delineated aquifer were also used to produce the vulnerability model. The final output of overlay parameters for estimating the groundwater potential gave an index that ranged from 1-5. The zone categorised as low groundwater potential covered about 80% of the area. The majority of the area falls within low (about 80%) vulnerability and low groundwater potential rating while being relatively protected from potential contaminants infiltrating from the surface. The prediction accuracy of the groundwater potential model was established via existing hand-dug well correlation analysis.

## 1 Introduction

Water plays a vital role in the socio economic development of any nation. This can be attributed basically to its importance in domestic and industrial uses. This relevance makes water a very viable resource and thus there is a need to explore for it in large quantity. Groundwater is the largest available source of fresh water (Venkateswaran et al., 2014). Groundwater is the most feasible alternative as the cost of exploitation via hand-dug well and boreholes is far cheaper when compared to conventional surface water programmes that will require construction of impounding reservoirs, piping network, etc. (Adeyeye et al., 2019). Comprehensive understanding of the groundwater system is necessary for sustainable development (Arsene et al., 2018). The availability of groundwater depends primarily on the geology. The Basement Complex is a heterogeneous mixture of crystalline rocks, predominantly granite or gneiss. The rocks in their pristine condition are inherently impermeable and contain negligible groundwater. Knowledge of the subsurface geology and structures are provided by geophysical surveys.

It is also imperative to note that, it is not enough to explore for availability of groundwater in terms of quantity alone. Emphasis should also be laid on the quality of the groundwater and how vulnerable the delineated aquifers are to contamination. In hydrogeology, vulnerability assessment typically describes the susceptibility of a particular aquifer to contamination that can reduce the groundwater quality (Al-Abadi et al., 2017). Vulnerability is the susceptibility of groundwater to contamination and it is a function of pollutant properties, anthropogenic activities, and physical parameters (Babiker et al. 2005).Vulnerability information can aid in the choice of proper locations for certain activities so that the adverse effects on groundwater are minimized, and protection of groundwater is achieved (Jamrah et al., 2008). Vulnerability assessment studies are used to identify areas that are more susceptible to contamination (Shahab et al., 2019). The susceptibility of the groundwater to pollutants is often accompanied by several factors with population growth and deficiency of surface storage facilities playing significant roles. These factors have consequently led to significant deterioration in both the quality and quantity of groundwater in the subsurface (Zghibi et al., 2016).

Geophysical methods have solved numerous exploration problems because it is rapid and can cover expanse of land in limited time and also can penetrate subsurface to a greater depth (Oladunjoye et al., 2019). Geoelectrical methods are particularly suitable for groundwater studies because the hydrogeological properties; such as porosity and permeability; can be correlated to electrical resistivity values (Helaly, 2017). Electrical resistivity method has been used successfully in delineation of hydrogeological zones for exploration of groundwater (Evans et al., 2010; George et al., 2010; Adiat et al., 2012; Ibuot et al., 2013; Akinlalu et al., 2017). Electrical resistivity method is one of the most useful methods in groundwater geophysics because the resistivity of rocks is sensitive to its ionic content. (Alile et al., 2011). The method allows a quantitative result to be obtained by using a controlled source of specific dimensions. The resistivity method is aimed at measuring the potential differences at the surface due to the current flow within the ground. Since the mechanisms that control the fluid flow and electric current and conduction are generally governed by the same physical parameters and lithological attributes, the hydraulic and electrical conductivities are dependent on each other (George et al., 2015). Geoelectric parameters derived from the electrical resistivity method assists in describing the hydrological condition of the subsurface and its aquifer protective capacity rating (Adeeko et al., 2019).

The study area Odode Idanre in southwestern Nigeria is a typical Basement Complex terrain and characterized by hills and mountains which makes access to potable water very difficult. Consequently, the groundwater development and management in the Basement Complex terrain requires a proper understanding of the hydrogeological characteristics of the aquifer units and the local geology (Abiola et al., 2009). This commonly necessitates a detailed geophysical investigation prior to groundwater exploitation to provide information on the subsurface lithology and aquiferous zones such as fractures, faults and joints that are favorable to groundwater accumulation and groundwater quality. However, geophysical investigation and other conventional techniques such as geostatistical and numerical modelling are often limited by lack of adequate data coverage for groundwater development and management (Jha et al., 2007).

A lot of study has been done in the area of groundwater potential evaluation and aquifer vulnerability in typical Basement Complex terrains around the globe (Omosuyi, 2010; Srinivasan et al., 2013; Zeyad, 2013; Akintorinwa and Olowolafe, 2013; Kamlesh & Shukla., 2014). These studies often applied geoelectrical and hydrogeological parameters such as aquifer resistivity, aquifer thickness, hydraulic conductance, transmittivity etc. independently for groundwater potential evaluation. Aquifer vulnerability studies usually involves the use of longitudinal conductance, ‘GOD’, ‘DRASTIC’ to mention a few (Oladapo et al., 2004; Foster et al., 2002; Mogaji et al., 2014). Also, the application of the concept of sensitivity analysis helps in determining the sensitivity of individual DRASTIC parameters to aquifer vulnerability (Colins et al., 2016). Khodadadi et al. (2015) and Khodabakhshi *et al.* (2017) respectively with great success evaluated the vulnerability of aquifers to pollution or contamination using modified GIS based methods and integration of GIS based DRASTIC and Groundwater quality index. However, integration of these factors will enhance our understanding on the susceptibility of the aquifers to contamination. Integration of different parameters will also assist in minimizing prediction error that may arise when geo-electric and hydrogeological parameters are used independently. The research therefore aims to develop a conceptual model for groundwater potential evaluation and aquifer vulnerability mapping in a typical Basement Complex environment using multi-criteria decision making analysis. Specific objectives are to delineate the subsurface layers and determine their geoelectric parameters, delineate possible geological features that are favourable for groundwater accumulation, delineate aquifer unit(s), determine their hydrogeological characteristics and produce the groundwater potential and aquifer vulnerability maps of the study area.

## 2 Study area

### 2.1 Location, accessibility and relief of the study area

**Figure 1:**
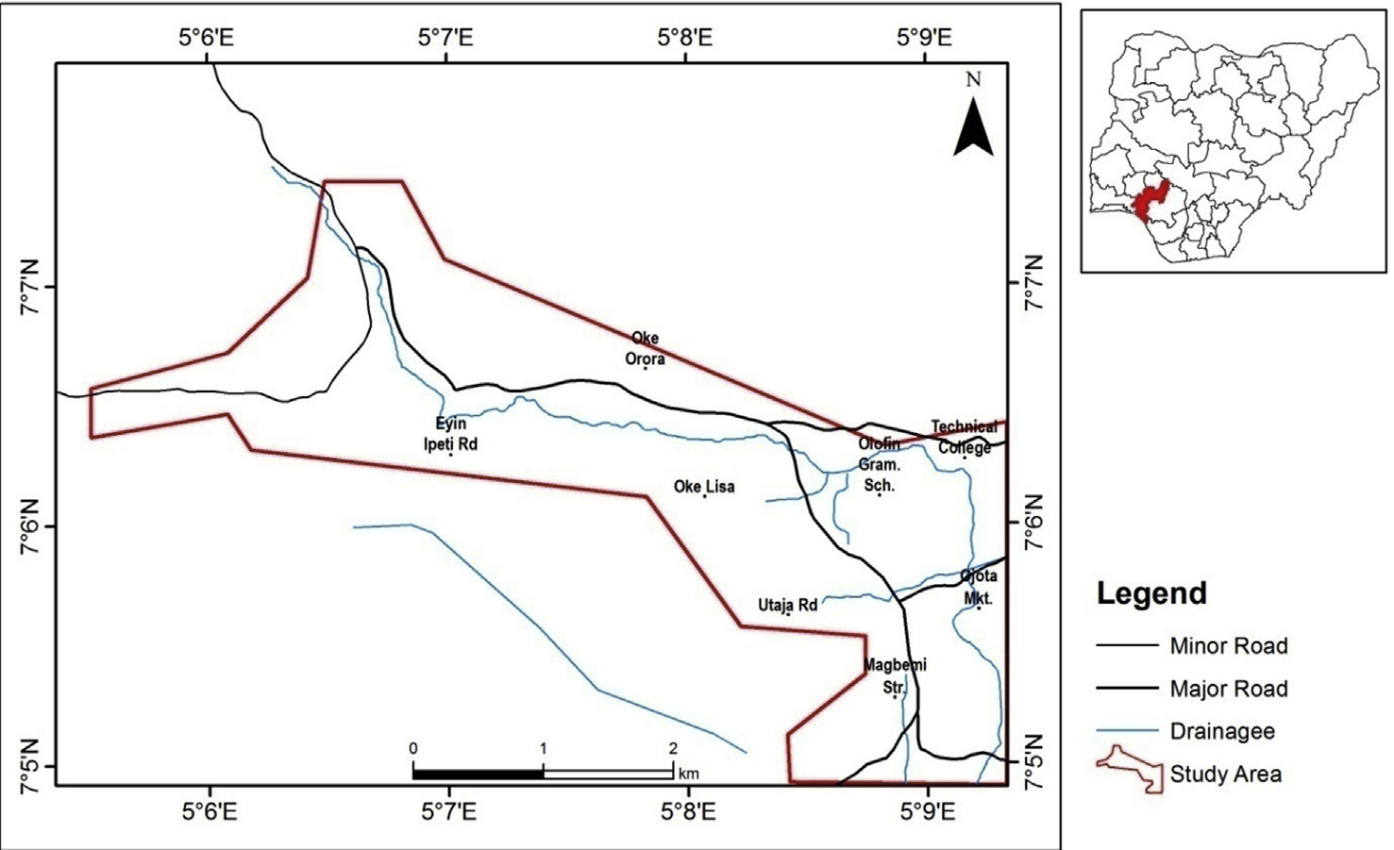
Location Map of the Study Area (Inset Map of Nigeria Showing Ondo State where Odode Idanre is located).

The study area is located at Odode Idanre, southwestern Nigeria. It covers areal extent of about 24.7 km^2^ and lies within 730000–737952 mE, and 782000–787464 mN in Universal Traverse Mercator (UTM) Zone 31N (Figure 1).

The entire area is generally accessible through a network of major and minor roads interconnecting the streets. The terrain is undulating with elevation ranging from 274 and 304 m above sea level. The main rain bearing wind affecting Odode Idanre is embedded in the easterly wind current (Owoyemi, 1996) as it lies within the tropical rain forest climate region of Nigeria.

### 2.2 Geology and hydrogeology of the study area

Idanre Hills is located on a Precambrian Igneous batholith that is about 500 Million years old, and is cut by several large fracture that form deep valleys within the rocks (Anifowose and Kolawole, 2012).

**Figure 2:**
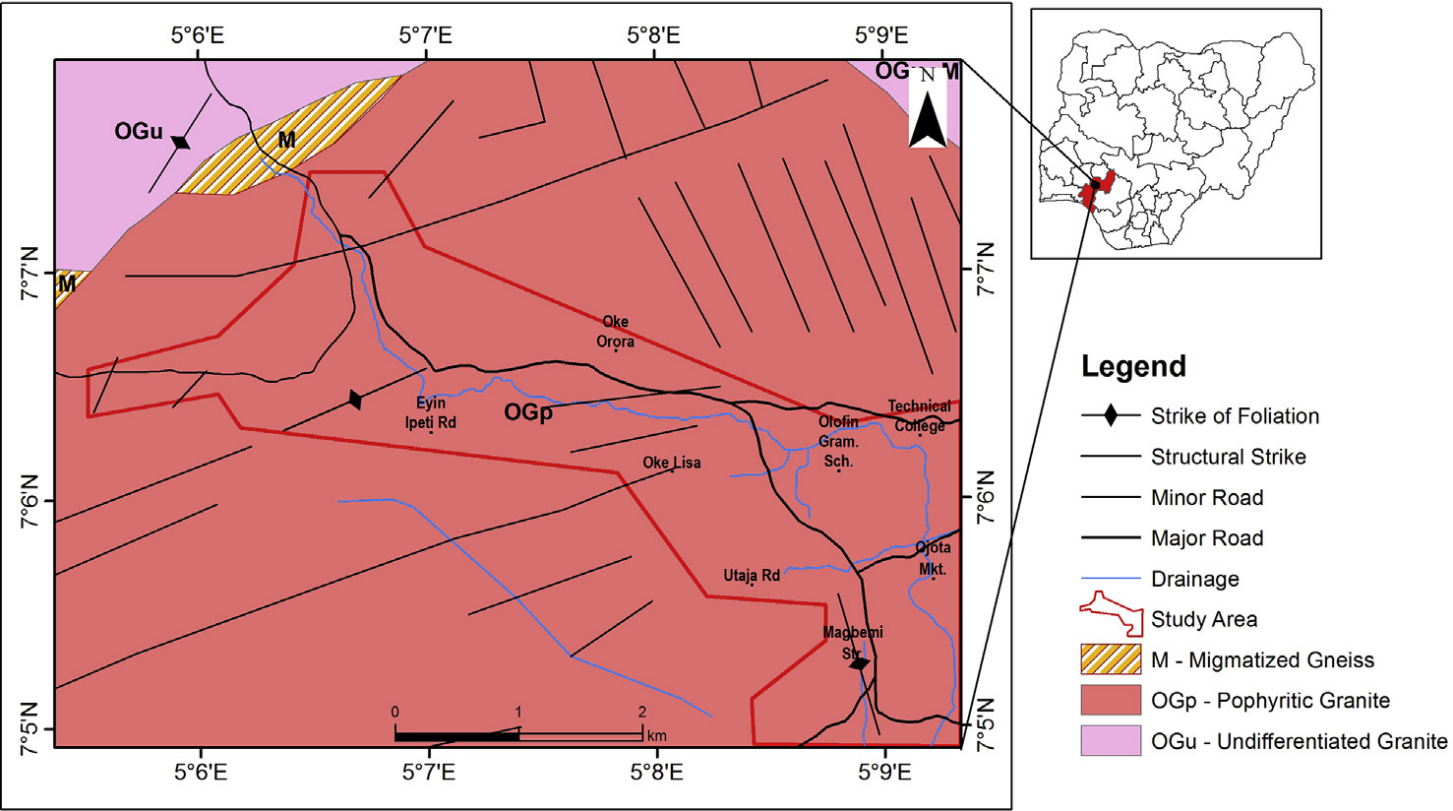
Geological Map of Idanre and its Environs Showing the Study Area.

Odode Idanre is underlain by three of the six major petrologic units of the Basement Complex described by Rahaman (1988). They are migmatite gneiss, members of the older granite and charnokitic rocks (Figure 2). Most of the outcrops observed in Odode Idanre are melanocratics, therefore possibly rich in biotite and/or hornblende. They show fine to medium grain textural characteristics. The light bands coloration observed on some of the smaller residual outcrops suggests that they are probably rich in feldspars and quartz. No visible surface structures except for a few short joint fissures generally trending North-South. Field observations show that granite rocks constitute extensive outcrops in perhaps over 80% of the study area (Ocan, 1991). According to Ocan (1991), granite gneisses are encountered in almost all outcrops either alone or in association with their components. The most common mode of occurrence is concordant or semi concordant bands, alternating with bands of grey gneiss or amphibolites. In some places, granite gneiss is seen to cross-cut the foliation of the grey gneiss. Xenoliths of the latter and the other mafic rocks are common in granite gneiss. This suggests that the granite gneiss is intrusive in origin and mineralogically, the granite gneiss in Idanre is composed of Alkali feldspar, quartz, plagioclase and biotite (Ocan, 1991). However, the areas of Odode Idanre that make up the study area are predominantly granitic rocks and these granitic rocks constitute extensive outcrops in perhaps about 20% of the study area. Also, the study area is made up of the Iwo soil association, characterized by coarse textured, sandy to fairly clayey soils. Therefore, the study area can be said to be monolithic in nature, which is of same geology (Figure 2) and soil composition.

In terms of hydrogeology, the Basement Complex area is mainly characterized by two major aquifer units, namely weathered and fractured Basement aquifers (Ako and Olorunfemi, 1989; Aniya and Shoeneick, 1992; Olorunfemi and Fasuyi, 1993; Afolayan et al., 2004 and Bayode et al., 2006). The Basement Complex rocks are mostly concealed by a sequence of unconsolidated superficial deposits and Basement regolith produced by prolonged weathering of the parent rock. Rocks dominated by unstable ferromagnesian minerals tend to weather into clay, sometimes, micaceous impermeable poor water discharging rock formations, while those rocks rich in quartz and other stable minerals will disintegrate into porous and permeable water bearing gravelly or sandy medium (Offodile, 2002).

The weathered layer aquifer may occur singly or in combination with the fractured aquifer. Olorunfemi and Fasuyi (1993) identified the aquifer combinations in the Basement Complex area as weathered layer aquifer; weathered/fractured (unconfined) aquifer; weathered/fractured (confined) aquifer; weathered/fractured (unconfined)/fractured (confined) aquifer and the fractured confined aquifer.

Porosity and permeability determine the hydrogeological properties of rocks and these characteristics depend on texture and mineralogy of rocks. In fresh, non-fractured crystalline rocks, the porosity is often less than 3 % and the permeability is virtually negligible. However, the porosity and permeability are increased considerably by weathering and fracturing (Offodile, 2002). Aquifers in the Basement rocks are highly limited in both horizontal and vertical extent.

## 3 Research methodology

**Figure 3:**
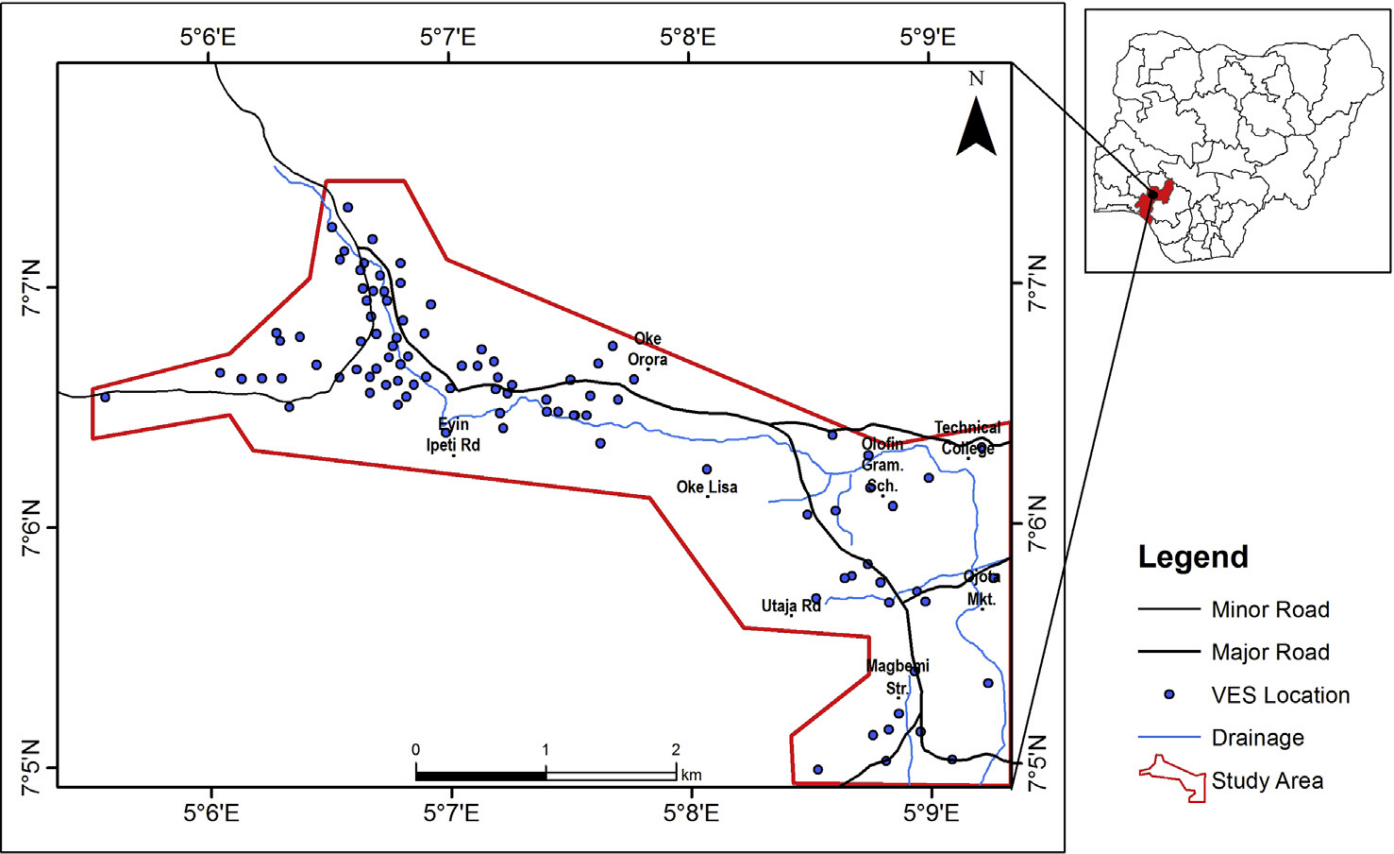
Data acquisition map of the study area.

**Figure 4:**
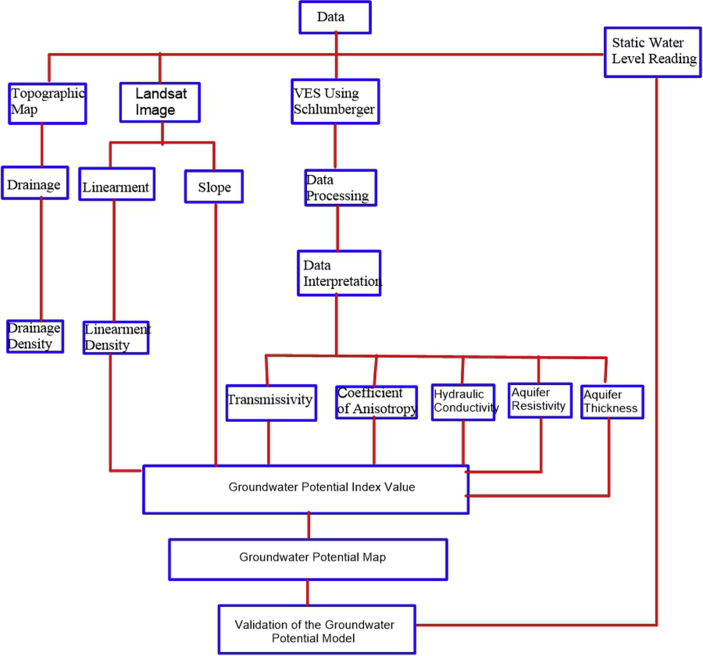
Methodology flow chart for groundwater potential evaluation.

**Figure 5:**
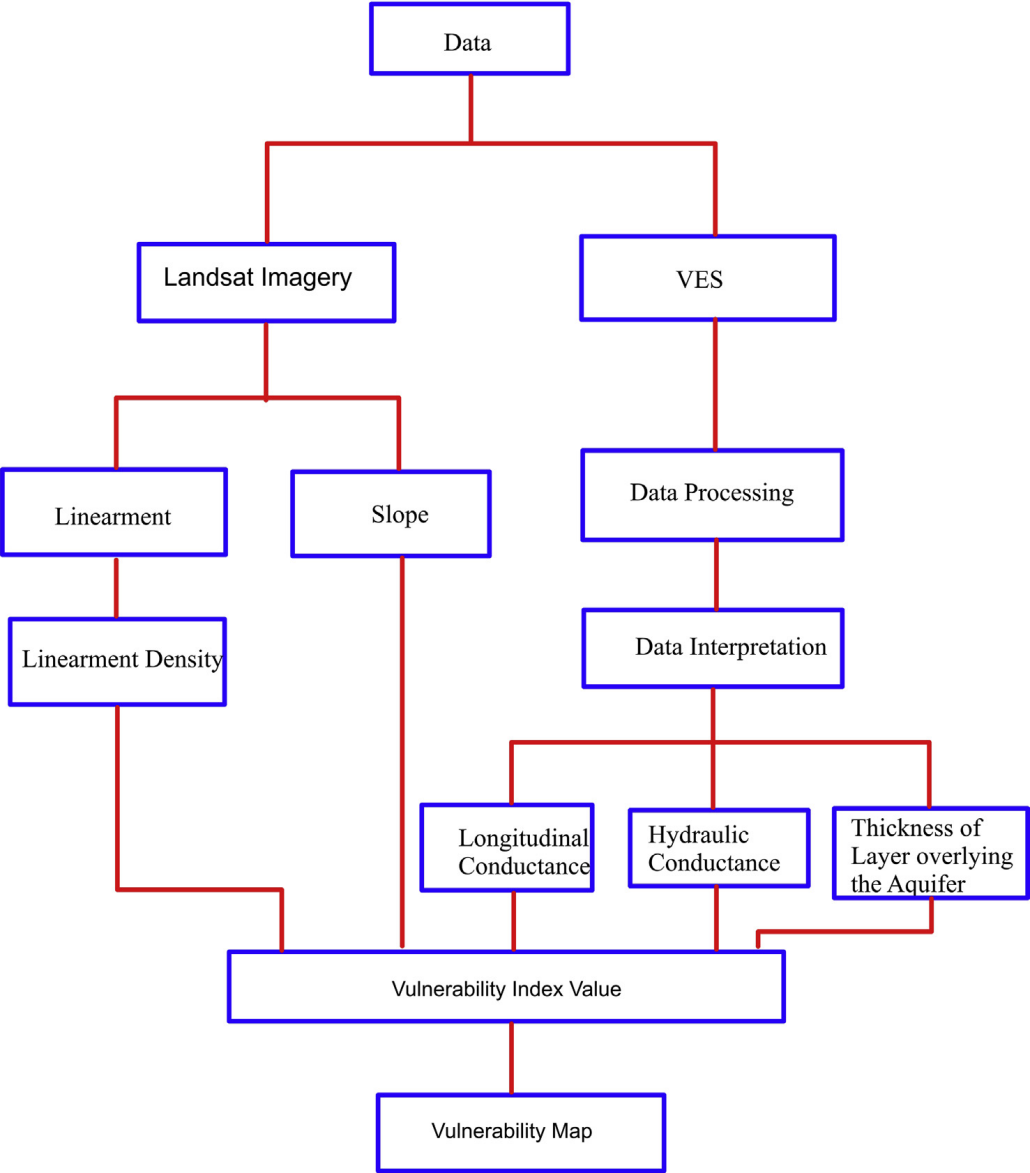
Methodology flow chart for vulnerability evaluation.

The research methodology was carried out in three phases which include remote sensing data analysis, geophysical investigation, and geographic information system (GIS) processing and interpretation. The data acquisition map and the methodology flow charts are presented in Figures 3, 4, and 5 respectively.

### 3.1 Remote sensing data

The remote Sensing involved obtaining the Landsat 7 thematic mapper imagery downloaded with path 189 and row 055 of the year 2006 from the Global Cover Facility homepage. The digital image processing methods used in this research work include radiometric, spatial and spectral enhancement techniques. The radiometric enhancement deals with an individual pixel of the image (Geosystem, 1999). Linear stretching as developed by Shanker (2007) was applied in this research. The linear stretching operation re-distributes the digital number (DN) values of an input map over a wider or narrower range of values in an output map which can be used to enhance the contrast in the image when it is displayed (Geosystems, 1999). The spatial enhancement technique modifies the DN value of the pixel based on the values of the surrounding pixels (ITC, 2001). Spectral enhancement techniques requires more than one spectral band, and involves data compression in order to reduce redundancy. The color composite was applied as multi band spectral enhancement techniques in this study. The RGB 321 was selected and used for the interpretation based on the representative of target on the imagery. Lineament and Slope were extracted from the Landsat image using the appropriate band combinations. The lineament density was subsequently obtained from the lineament map.

Lineament mapping was carried out on the Landsat image bands through Principal Component Analysis (PCA), Image Enhancement and Visual Interpretation.

Four standard gradient filters in earth resources data analysis system using ERDAS IMAGINE™ which enhance linear features in different directions were employed in this work (Erdas Inc., 2001). These directions are N-S, E-W, NE-SW and NW-SE. The enhanced linear features were then digitized by visual interpretation. Lineament map was generated using ArcGIS 10.1™ software (ESRI, 2001). The lineament density was calculated using the Line Density Tool in the GIS environment by adopting Eq. (1).

(1) $$\text{Lineament density}\left(l\right)=\frac{\text{Total Lineament Length}\left(\text{L}\right)}{\text{Study area}\left(\text{A}\right)}$$

Drainage density can be defined as the ratio of the total stream length to the total drainage area. In order to derive this, first the drainage pattern of the study area was mapped. The drainage pattern of the study area was obtained from the Digital Elevation Model (DEM) by filling the sinks in DEM. This was done in order to remove some imperfections in the image.

The drainage density (Equation 2) was calculated using the Line Density Tool in ArcMap within the ArcGIS software.

(2) $$\text{Drainage density}\left(\partial \right)=\frac{\text{Total Stream Length}\left(\text{L}\right)}{\text{Drainage area}\left(\text{A}\right)}$$

Slope is the gradient or rate of maximum change of the terrain. The slope map was generated from the triangulated irregular network (TIN) that was obtained from the elevation surface values through interpolation using the processed Landsat Imagery.

### 3.2 Ancillary data

Topographic and geological map of the study area were obtained from the Federal Survey of Nigeria (FSN) and Nigeria Geological Survey Agency (NGSA) respectively. The topographic map assisted in extracting the drainage and subsequently the drainage density map of the area. The maps were digitized for better resolution.

### 3.3 Geophysical investigation

#### 3.3.1 Data collection

One hundred and one (101) Vertical Electrical Soundings (VES) data were acquired in the study area, using the Schlumberger array, with half current electrode spacing (AB/2) varying from 1 to 100 m (Figure 3). The Ohmega resistivity meter was used for data acquisition. The position of the occupied sounding stations in Universal Traverse Mercator (UTM) was recorded using Geographic Positioning System (GPS) unit. The sounding data were acquired along roads, linear routes between houses and any other available open spots. The Vertical Electrical Sounding (VES) data were processed by calculating the apparent resistivity $\left(\rho_{a}\right)$ values from the product of the resistance (R) obtained from the equipment and the geometrical factors (G) of the respective electrode separation for each spread length.

The calculated apparent resistivity $\left(\rho_{a}\right)$ values at each of the VES stations were plotted against electrode spacing (AB/2) on the bi-logarithm graph sheet. The resulting curves were qualitatively interpreted by visual inspection of the curves to determine the nature of the subsurface layer. Partial curve matching was carried out on the generated field curves for the quantitative interpretation. The result of the curve matching (layers resistivity and thickness) were fed into computer as starting model parameter in an iterative forwarded modeling technique using WinResist Software (Vander-Velpen, 2004). The geoelectric parameters obtained (Resistivity, Thickness and Depth) assisted in identifying the aquifer thickness and aquifer resistivity which are important factors in groundwater potential evaluation (Table 1).

**Table 1:** Summary of VES interpretation results.

VES	Layer Thickness (m)				Layer Resistivity (ohm-m)					Curve Type
	h_1_	h_2_	h_3_	h_4_	ρ_1_	ρ_2_	ρ_3_	ρ_4_	ρ_5_	
1	1	5.1			22	189	3242			A
2	0.5	1.2	3.9		24	630	86	∞		KH
3	0.6	2.1	3.2		41	90	∞			A
4	1.8	1.2			41	90	∞			A
5	0.9	1.7			99	159	∞			A
6	1.3	1			38	96	∞			A
7	1.1	2.4			33	23	∞			H
8	0.5	5.8			22	197	2522			A
9	0.4	1.1	2.3		35	139	14	∞		KH
10	1.2	0.5	3		88	25	49	4981		HA
11	0.5	0.5	2.1		114	300	103	5682		KH
12	0.8	1.1			31	284	4213			A
13	0.5	1.3	2.8		198	23	480	∞		HA
14	0.7	0.6	2.6		84	157	72	8419		KH
15	0.4	0.9			256	22	∞			H
16	0.3	0.5	1.1		38	115	18	9343		KH
17	0.4	2.8			35	03	∞			H
18	0.5	1.2	7.4		94	44	85	∞		HA
19	0.8	1.6			41	25	6149			H
20	0.6	1.9			142	97	∞			H
21	0.5	0.7	3.7		20	104	28	∞		KH
22	0.6	3			42	32	∞			H
23	1.6	3.7	5		165	46	669	∞		HA
24	1.6	22.5			58	236	1184			A
25	1	2.1			79	26	9233			H
26	1.5	2.2	1.7		60	38	351	1205		HA
27	0.4	0.6	3		22	119	23	∞		KH
28	1.3	5.8			170	118	∞			H
29	0.4	0.6	7.7		67	109	24	7251		KH
30	2.6	18.7			36	1853	3479			A
31	0.5	0.9			13	891	3172			A
32	0.5	0.9	4.5		33	105	31	∞		KH
33	0.8	2.7	4.1		63	71	874	∞		AA
34	2.3	6.7			26	221	1553			A
35	0.4	12.4			233	123	1357			H
36	0.5	3.7	5.9		127	457	204	∞		KH
37	0.5	4.2			61	70	3100			A
38	0.6	1.6			73	54	1198			H
39	0.7	1.8	20.9		46	73	254	∞		AA
40	1	2.2			153	23	∞			H
41	1	1.6			76	47	∞			H
42	1.8	14			60	27	599			H
43	1	3.2			88	84	9868			H
44	0.5	7.7			66	159	∞			A
45	0.5	5.2			64	79	8283			A
46	0.5	3.8	10.1		48	88	864	52		AK
47	1.6	5.7			67	124	∞			A
48	0.7	2.2			414	411	4808			H
49	1	1.7			169	234	1637			A
50	0.5	9.8			830	3035	5591			A
51	1.2	14.5			39	173	5011			A
52	0.9	1.6			40	15	2193			H
53	1.1	1.9			105	280	∞			A
54	1	6.6			61	150	8121			A
55	1.4	1.2	1.5		52	19	239	4289		HA
56	0.7	2.3			48	154	1799			A
57	2.9	2.3			44	475	∞			A
58	0.4	3	5		51	114	706	∞		AA
59	0.8	6.9			67	87	2104			A
60	3.8	2.9			48	175	3352			A
61	1.4	11.6			33	198	8562			A
62	0.9	1.7			75	97	∞			A
63	0.7	4.3			41	186	∞			A
64	0.8	3.8	5.5		95	70	713	∞		HA
65	0.5	3.4			411	15	6402			H
66	1.2	0.9	3.5		79	158	40	∞		KH
67	1	0.1			41	2000	∞			A
68	2.2	5.2			98	52	∞			H
69	1.1	1.6			40	05	∞			H
70	1.7	4.6	37		56	125	49	∞		KH
71	1	5.1			118	98	∞			H
72	2.1	2.8			34	41	∞			A
73	1.9	6.9			70	182	2181			A
74	1.6	5.5			111	83	∞			H
75	1	0.4			114	1353	∞			A
76	1.2	2.4			60	35	∞			H
77	1.1	3.7			66	76	1244			A
78	1.8	20.7			44	174	∞			A
79	0.9	2.2			110	56	∞			H
80	2.5	6			217	17	∞			H
81	1.5	1.6	0.5	1.4	32	24	365	05	∞	HKH
82	2.2	11			37	144	1715			A
83	2.1	3.9			43	71	2689			A
84	1.2	0.8			130	30	566			H
85	1.3	7.8			132	30	∞			H
86	1.8	21			122	540	1171			A
87	1.1	0.6	4.6	32	45	31	3529	320	2487	HKH
88	1.2	0.4	5.4		63	188	57	∞		KH
89	1.5	5.2			70	237	2547			A
90	1	2.1			118	131	976			A
91	0.7	28.7			89	270	∞			A
92	0.9	6.5			47	704	∞			A
93	0.5	2.2	1.7	13.9	50	14	810	43	∞	HKH
94	1.1	1.6			30	17	∞			H
95	1.5	5			75	48	∞			H
96	1	17.7			65	429	945			A
97	0.9	13.7			72	251	∞			A
98	1	1.6	16.4		129	61	141	∞		HA
99	1	10.6			49	176	∞			A
100	1.1	1.4	6		78	6018	183	1926		KH
101	1.3	6			73	295	1154			A

#### 3.3.2 Second order geo-electric parameters

The first order parameters (geoelectric parameters) (Table 1) were used to determine the second order parameters i.e Longitudinal Conductance (S), Transverse Resistance (T), Longitudinal Resistivity (ρ_L)_ and Transverse Resistivity (ρt) as presented in Eqs. (3) and (4).

(3) Longitudinalunitconductance,S=h/ρ=hσ

For "n" layers, the total longitudinal unit conductance is:

(4) S = h_1_/ρ_1_ + h_2_/ρ_2_ + …… + h_n_/ρ_n_

where S = Longitudinal Unit Conductance, h = layer thickness, ρ = layer resistivity, σ = conductivity, n = nth layer (1,2………..n).

The coefficient of Anisotropy (λ) was calculated by substituting the longitudinal resistivity (ρ_L_) and transverse resistivity (ρt) into Eq. (5). The results of the calculated second order parameters were used to generate longitudinal conductance and coefficient of Anisotropy maps which play significant roles in aquifer vulnerability and groundwater assessment respectively.

The hydraulic conductivity (K) and aquifer transmissivity (T_t_) were estimated using Eqs. (6) and (7) as presented by Odong (2013).

(5) $$\lambda =\sqrt{\frac{\rho_{t}}{\rho_{L}}}$$

where λ is coefficient of anisotropy; ρt = transverse resistivity and ρ_L_ = Longitudinal resistivity

(6) $$\text{K}=0.0538{\text{e}}^{-0.0072\text{ρ}}$$

where:

K = Hydraulic Conductivity m/s

ρ = Apparent Resistivity

(7) T=Kb

where:

T = Transmissivity m^2^/s

K = Hydraulic Conductivity

b = Aquifer Thickness

From the estimated K and T, the aquifer unit hydraulic conductivity and Transmissivity maps of the study area were produced.

Applications of Eqs. (3), (4), (5), (6), and (7) on the geo-electric parameters on Table 1 assisted in obtaining the second order parameters presented in Table 2.

**Table 2:** Determined groundwater potentiality and vulnerability influencing parameters.

VES NO	COA (Ω)	LC Mhos	AT (m)	AR (Ωm)	TLOA (Ωm)	K m/s	T m^2^/day	LD Km^2^	Slope (degree)	DD (Km^2^)	GWPI	VI
1	1.3854	0.0724	5.1	189	1.0	0.0138	0.0120	0.0000	2.9698	2.3083	100	100
2	1.5478	0.0681	3.9	86	1.7	0.0290	0.0504	0.0000	4.1449	1.6436	125	140
3	1.1572	0.1426	3.2	31	2.7	0.0430	0.1030	0.0000	4.4075	3.4163	150	220
4	1.0753	0.0572	1.2	90	1.8	0.0281	0.0145	0.0000	13.8968	3.1975	125	140
5	1.0256	0.0198	1.7	159	0.9	0.0171	0.0066	0.0000	8.2975	3.1991	100	100
6	1.1075	0.0446	1.0	96	1.3	0.0270	0.0110	0.0000	3.8364	2.7243	125	140
7	1.0140	0.1377	2.4	23	1.1	0.0456	0.0881	0.0000	1.8562	2.8104	150	180
8	1.2313	0.0522	5.8	197	0.5	0.0130	0.0119	0.0000	13.5442	3.6521	125	100
9	1.5911	0.1836	2.3	14	1.5	0.0486	0.0981	0.0000	42.9566	2.5742	200	260
10	1.0670	0.0949	3.0	49	1.7	0.0378	0.0716	0.0000	5.5723	3.4645	150	140
11	1.0791	0.0264	2.1	103	1.0	0.0256	0.0205	0.0000	16.8934	2.6887	125	140
12	1.6650	0.0297	1.1	284	0.8	0.0070	0.0005	0.0000	6.3140	2.5956	100	100
13	2.1251	0.0649	2.8	480	1.8	0.0017	0.0001	0.0000	12.0317	2.3601	150	100
14	1.0390	0.0483	2.6	72	1.3	0.0320	0.0424	0.0000	25.6386	0.0000	150	180
15	1.7524	0.0425	0.9	22	0.4	0.0460	0.0336	0.0000	32.2288	0.2741	175	220
16	1.3425	0.0734	1.1	18	0.8	0.0473	0.0439	0.0000	13.4032	4.8530	175	180
17	1.4376	0.9448	2.8	3	0.4	0.0527	0.1433	0.0000	13.2226	3.5505	175	260
18	1.0262	0.1197	7.4	85	1.7	0.0292	0.0973	0.0000	17.3670	0.2194	125	140
19	1.0274	0.0835	1.6	25	0.8	0.0449	0.0569	0.0000	11.2707	0.2166	150	180
20	1.0133	0.0238	1.9	97	0.6	0.0268	0.0205	0.0000	5.0085	5.0165	150	140
21	1.1279	0.1639	3.7	28	1.2	0.0440	0.1251	0.0000	9.3168	5.4521	175	180
22	1.0052	0.1080	3.0	32	0.6	0.0427	0.0950	0.0000	3.5461	6.3133	175	180
23	1.8647	0.0977	5.0	669	5.3	0.0004	0.0000	0.0000	3.5461	3.0743	150	180
24	1.0693	0.1229	22.5	236	1.6	0.0098	0.0242	0.0000	2.0752	1.8488	125	100
25	1.1397	0.0934	2.1	26	1.0	0.0446	0.0734	0.0000	6.0127	1.2260	150	180
26	1.5224	0.0877	1.7	351	3.7	0.0043	0.0003	0.0000	3.9600	0.0000	125	140
27	1.1970	0.1537	3.0	23	1.0	0.0456	0.1102	0.0000	4.3094	0.0000	150	180
28	1.0100	0.0568	5.8	118	1.3	0.0230	0.0441	0.0000	4.9226	0.0000	125	140
29	1.1028	0.3323	7.7	24	1.0	0.0453	0.2783	0.0000	2.3657	0.0000	150	180
30	2.5107	0.0823	0.0	0	0.0	0.0000	0.0000	0.0000	2.3198	2.4783	75	60
31	4.0349	0.0395	0.9	891	0.5	0.0001	0.0000	0.0000	3.3437	2.1035	200	100
32	1.1024	0.1689	4.5	31	1.4	0.0430	0.1448	0.0000	1.3532	1.0956	150	180
33	1.9158	0.0554	4.1	874	3.5	0.0000	0.0000	0.0000	4.3094	0.0000	175	140
34	1.5030	0.1188	6.7	221	2.3	0.0110	0.0092	0.0000	2.3198	2.6302	100	100
35	1.0064	0.1025	12.4	123	0.4	0.0222	0.0868	0.0000	4.5857	2.4864	125	140
36	1.0898	0.0410	5.9	204	4.2	0.0124	0.0108	0.0000	3.9464	2.1299	100	140
37	1.0009	0.0682	4.2	70	0.5	0.0325	0.0708	0.0000	8.4970	0.0000	125	140
38	1.0090	0.0378	1.6	54	0.6	0.0365	0.0352	0.0000	14.5418	2.1237	125	140
39	1.1049	0.1222	20.9	254	2.5	0.0086	0.0167	0.0000	4.2720	2.4794	125	140
40	1.4254	0.1022	2.2	23	1.0	0.0456	0.0808	0.0000	5.3282	2.6985	150	180
41	1.0275	0.0472	1.6	47	1.0	0.0384	0.0395	0.0000	3.1110	2.5975	125	140
42	1.0334	0.5485	14.0	27	1.8	0.0443	0.4814	0.0000	16.3615	2.1267	187.5	220
43	1.0002	0.0495	3.2	84	1.0	0.0294	0.0428	0.0000	17.3779	0.0000	125	140
44	1.0233	0.0560	7.7	159	0.5	0.0171	0.0297	0.0000	10.0091	0.0000	100	100
45	1.0018	0.0736	5.2	79	0.5	0.0305	0.0755	0.0000	14.3501	0.0000	125	140
46	1.6913	0.0653	0.0	0	0.0	0.0538	0.0000	0.0000	6.2887	0.0000	112.5	160
47	1.0329	0.0698	5.7	124	1.6	0.0220	0.0393	0.0000	4.5624	0.0000	125	140
48	1.0000	0.0070	2.2	411	0.7	0.0028	0.0001	0.0000	32.9523	0.5409	150	140
49	1.0124	0.0132	1.7	234	1.0	0.0100	0.0019	0.0000	10.4086	1.5952	100	100
50	1.0436	0.0038	0.0	0	0.0	0.0000	0.0000	0.0000	9.4387	1.3349	50	60
51	1.0899	0.1146	14.5	173	1.2	0.0155	0.0443	0.0000	6.2123	2.6332	125	100
52	1.1136	0.1292	1.6	15	0.9	0.0483	0.0671	0.0000	6.1695	3.4191	150	180
53	1.1144	0.0173	1.9	280	1.1	0.0072	0.0010	0.0000	6.0127	2.6482	100	100
54	1.0483	0.0604	6.6	150	1.0	0.0183	0.0295	0.0000	19.7931	2.3343	125	140
55	1.6134	0.0964	1.5	239	2.6	0.0096	0.0015	0.0000	13.5442	0.0000	100	100
56	1.1278	0.0295	2.3	154	0.7	0.0178	0.0096	0.0000	2.4983	1.7952	100	100
57	1.7867	0.0708	2.3	475	2.9	0.0018	0.0000	0.0000	5.3282	0.0000	125	100
58	1.5083	0.0412	5.0	706	3.4	0.0003	0.0000	0.0000	0.3283	1.8219	150	140
59	1.0032	0.0913	6.9	87	0.8	0.0288	0.0877	0.0000	1.3532	0.0000	125	140
60	1.2130	0.0957	2.9	175	3.8	0.0153	0.0086	0.0000	0.7340	3.5206	125	140
61	1.1834	0.1010	11.6	198	1.4	0.0129	0.0234	0.0000	0.7340	2.6194	100	100
62	1.0075	0.0295	1.7	97	0.9	0.0268	0.0183	0.0000	1.1834	2.6194	125	140
63	1.1541	0.0402	4.3	186	0.7	0.0141	0.0106	0.0000	1.4677	2.5734	100	100
64	1.7156	0.0704	5.5	713	4.6	0.0003	0.0000	0.0000	0.0000	2.6349	150	140
65	1.9604	0.2279	3.4	15	0.5	0.0483	0.1426	0.0000	4.0535	3.3402	175	180
66	0.6456	0.0347	3.5	40	2.1	0.0403	0.0970	0.0000	7.0352	3.8346	175	180
67	2.2063	0.0244	0.0	0	0.0	0.0000	0.0000	0.0000	10.6095	1.9193	75	60
68	1.0425	0.1224	5.2	52	2.2	0.0370	0.1181	0.0000	2.2009	4.4481	150	140
69	1.5744	0.3475	1.6	5	1.1	0.0519	0.0792	0.0000	12.2861	2.8841	150	220
70	1.0436	0.8223	36.6	49	6.3	0.0378	0.8739	0.0000	5.0085	6.0823	250	300
71	1.0024	0.0605	5.1	98	1.0	0.0266	0.0540	0.0000	8.6916	0.0000	125	140
72	1.0043	0.1301	2.8	41	2.1	0.0400	0.0763	0.0000	7.3362	1.9864	150	180
73	1.0801	0.0651	6.9	182	1.9	0.0145	0.0182	0.0000	10.0091	4.4568	125	100
74	1.0074	0.0807	5.5	83	1.6	0.0296	0.0747	0.5782	1.3128	1.9384	125	140
75	1.7410	0.0091	0.0	0	0.0	0.0000	0.0000	0.0000	3.4230	2.8320	50	60
76	1.0325	0.0886	2.4	35	1.2	0.0418	0.0723	0.2043	0.4642	3.6700	175	180
77	1.0018	0.0654	3.7	76	1.1	0.0311	0.0565	0.0000	1.6733	4.0026	150	140
78	1.0782	0.1599	20.7	174	1.8	0.0154	0.0622	0.0000	2.3883	3.4176	125	100
79	1.0476	0.0475	2.2	56	0.9	0.0360	0.0468	0.0000	5.3282	0.0000	125	140
80	1.8031	0.3645	6.0	17	2.5	0.0476	0.2435	0.8748	1.3128	6.0355	225	300
81	2.6924	0.3949	1.4	5	3.6	0.0519	0.0693	0.2607	5.2880	3.0158	175	260
82	1.1395	0.1358	11.0	144	2.2	0.0191	0.0544	0.4602	3.6650	2.0276	100	100
83	1.0288	0.1038	3.9	71	2.1	0.0323	0.0647	0.0000	1.6408	2.6699	125	140
84	1.2710	0.0359	0.8	30	1.2	0.0433	0.0262	0.6613	2.6446	0.9318	150	180
85	1.1497	0.2698	7.8	30	1.3	0.0433	0.2552	0.0000	7.4432	2.0363	150	180
86	1.0922	0.0536	21.0	540	1.8	0.0011	0.0001	0.0000	4.7234	3.0954	150	100
87	1.6203	0.1451	0.6	31	1.1	0.0430	0.0193	1.2032	2.0752	2.3807	175	220
88	2.1786	0.1159	5.4	57	1.6	0.0357	0.1129	0.0000	4.3094	0.0000	150	140
89	1.1367	0.04337	5.2	237	1.5	0.0098	0.0055	0.0000	14.3880	4.5743	125	100
90	1.0012	0.0245	2.1	131	1.0	0.0209	0.0129	0.0000	6.6649	1.1638	125	140
91	1.0157	0.1142	28.7	269	0.7	0.0078	0.0179	0.0000	0.0000	2.6551	150	100
92	1.5471	0.0284	6.5	704	0.9	0.0003	0.0000	0.0000	4.1449	2.7731	150	100
93	1.7280	0.4925	13.9	43	4.4	0.0395	0.3666	0.4386	5.0825	1.9671	200	280
94	1.0392	0.1308	1.6	17	1.1	0.0476	0.0649	0.0000	1.0379	2.5827	150	180
95	1.0179	0.1242	5.0	48	1.5	0.0381	0.1214	0.6951	3.6209	1.8944	125	140
96	1.1138	0.0566	17.7	429	1.0	0.0025	0.0008	0.3876	3.0937	2.4028	150	100
97	1.0500	0.0671	13.7	251	0.9	0.0088	0.0115	0.0000	0.0000	0.0000	125	100
98	1.0281	0.1503	16.4	141	2.6	0.0195	0.0852	0.0000	0.0000	0.0000	125	140
99	1.0711	0.0806	10.6	176	1.0	0.0152	0.0308	1.3280	1.7671	0.0000	125	140
100	2.5034	0.0471	6.0	183	1.5	0.0144	0.0155	1.3238	2.2009	1.8483	150	140
101	1.1554	0.0381	6.0	295	1.3	0.0064	0.0024	0.0000	3.0235	0.0000	100	100

COA = Coefficient of Anisotropy; LC = Longitudinal Conductance; AT = Aquifer Thicknesses.

AR = Aquifer Resistivity; TLOA = Thickness of Layer Overlying the Aquifer.

T = Transmissivity; K = Hydraulic Conductivity; S = Slope; LD = Lineament Density; DD = Drainage Density, GWPI = Groundwater Potential Index, VI = Vulnerability Index.

#### 3.3.3 Selected factors for groundwater potential evaluation

In all, Eight (8) factors were considered for the evaluation of groundwater potential in the study area. The factors are drainage density, lineament density, slope, transmissivity, hydraulic conductivity, coefficient of anisotropy, aquifer resistivity and aquifer thickness.

#### 3.3.4 Selected factors for aquifer Vulnerability Evaluation

Four (4) factors namely lineament density, longitudinal conductance, hydraulic Conductance and thickness of layer overlying the aquifer were considered in evaluating how vulnerable the aquifer is to pollution or contamination.

### 3.4 Geographic information system (GIS) technique

Maps for the geophysical parameters were generated using ArcGIS 10.1 software (2010). These were done using the inverse distance weighting (IDW) method.

### 3.5 Multi-criteria modeling

#### 3.5.1 Groundwater potential index (GWPI) and vulnerability index (VI) estimation

The GWPI and VI estimation were obtained by relating the weights and ratings of the contributing parameters to groundwater potentiality and vulnerability mapping as presented in Eqs. (8) and (9). On the ArcGIS platform, weighted linear average was applied to carry out the estimation.

(8) GWPI=∑WiRi

(9) VI=∑WiRi

where W is the weight (W) of parameter ‘i’ and R is the rating score of parameter ‘i’.

#### 3.5.2 Preparation of groundwater potential and aquifer vulnerability models

The groundwater potential index (GWPI) and vulnerability index (VI) and the parameters through which they were derived are as shown in Table 2. They were estimated by substituting the rating factors of each considered parameters into Eqs. (8) and (9) respectively. The groundwater potential index (GWPI) and vulnerability index (VI) obtained at each of the VES stations were used to produce the groundwater potential and vulnerability model using ArcGIS software.

#### 3.5.3 Weighted overlay

The Weighted overlay was achieved by the assignment of equal weights to all the parameters involved in each analysis.

For the Groundwater Potential, (8) eight parameters were involved with 12.5% weight for each parameter, while for the Vulnerability (5) five parameters were involved with 20 % weight for each parameter. Values for each parameter are reclassified into three classes scale of 5, 3 and 1. 5 indicating higher potential and vulnerability, 3 moderate while 1 to a lower potential and vulnerability. The maps were overlaid by the product of each location suitability value with its layer weight. The total values were used to derive and generate composite maps of Groundwater Potential and Vulnerability.

#### 3.5.4 Validation of results

The objective of the validation is to check if the prediction accuracy of groundwater model gives reality of the produced groundwater potential model. The validation was done by taking the inventory of forty-eight (48) hand-dug wells distributed across the study area. This was done between 3^rd^ and 4th February, 2018 (dry season). The relationship between the produced groundwater potential map and the volume of the well as obtained from the static water level and water column level assist in validating the output of this study. The water column level of each well was estimated. The water column level was obtained by subtracting the static water level from the well depth (Akinlalu et al., 2017).

## 4 Results and discussions

### 4.1 Discussions on factors controlling groundwater potential mapping

#### 4.1.1 Drainage density map

**Figure 6:**
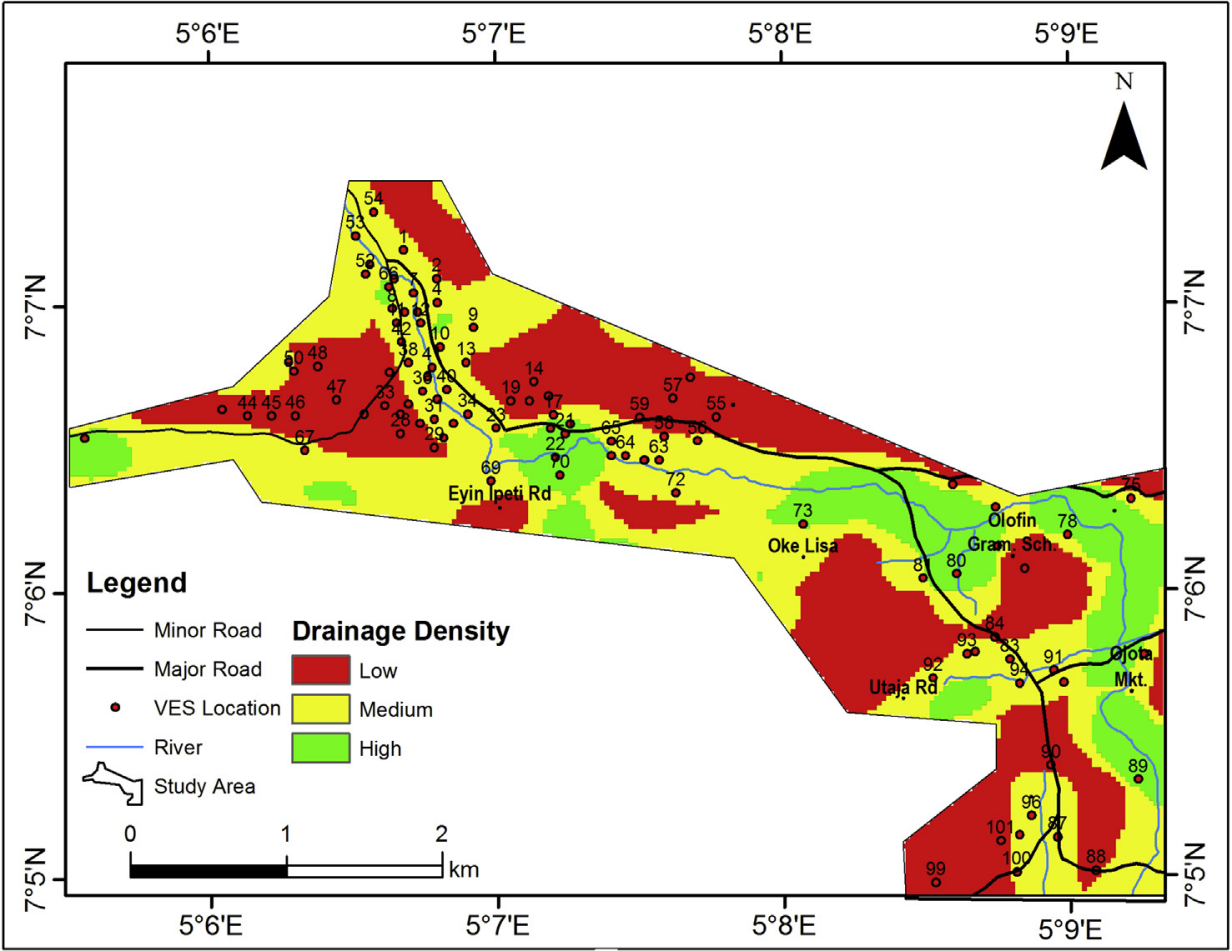
Drainage density map of the study area.

The drainage density map of the study area (Figure 6) reveals the density of the drainage network as a measure of the closeness of spacing of the stream segments of all orders per unit area. The drainage density of the study area was grouped into three classes; 0.00–1.37 per km^2^, 1.38–3.78 per km^2^ and 3.79–9.17 per km^2^ which indicate low, medium and high drainage density respectively. The areas characterized by high drainage density correspond to the pattern of the major ambient river channels in the study area. However, the groundwater potential is indirectly related to the drainage density of an area as the latter is mostly a product of good run-off and/low permeability (Magesh et al., 2012).

#### 4.1.2 Lineament density map of the study area

**Figure 7:**
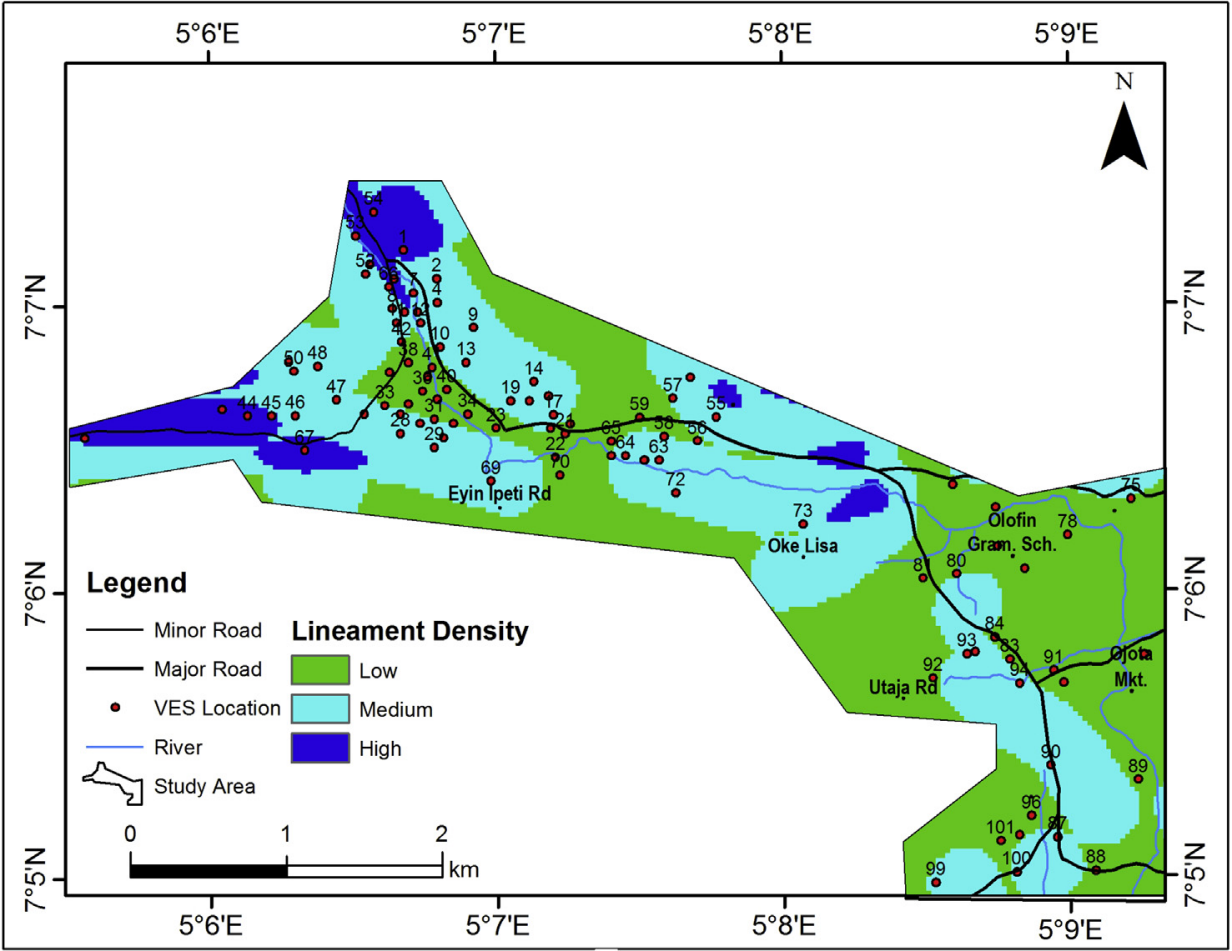
Lineament density map of the study area.

The occurrence of lineament is directly proportional to the groundwater potential and aquifer vulnerability of an area, since lineaments represent the zones of faulting and fracturing resulting in increased secondary porosity and permeability. The lineament density map of the study area (Figure 7) were grouped into three classes; 0.00–0.37 per km^2^, 0.38–1.11 per km^2^ and 1.12–1.92 per km^2^ indicating low, medium and high lineament density respectively. Low lineament density dominates the study area. Based on the predominant low lineament density, the study area may be rated as of low groundwater potential.

#### 4.1.3 Slope of the study area

**Figure 8:**
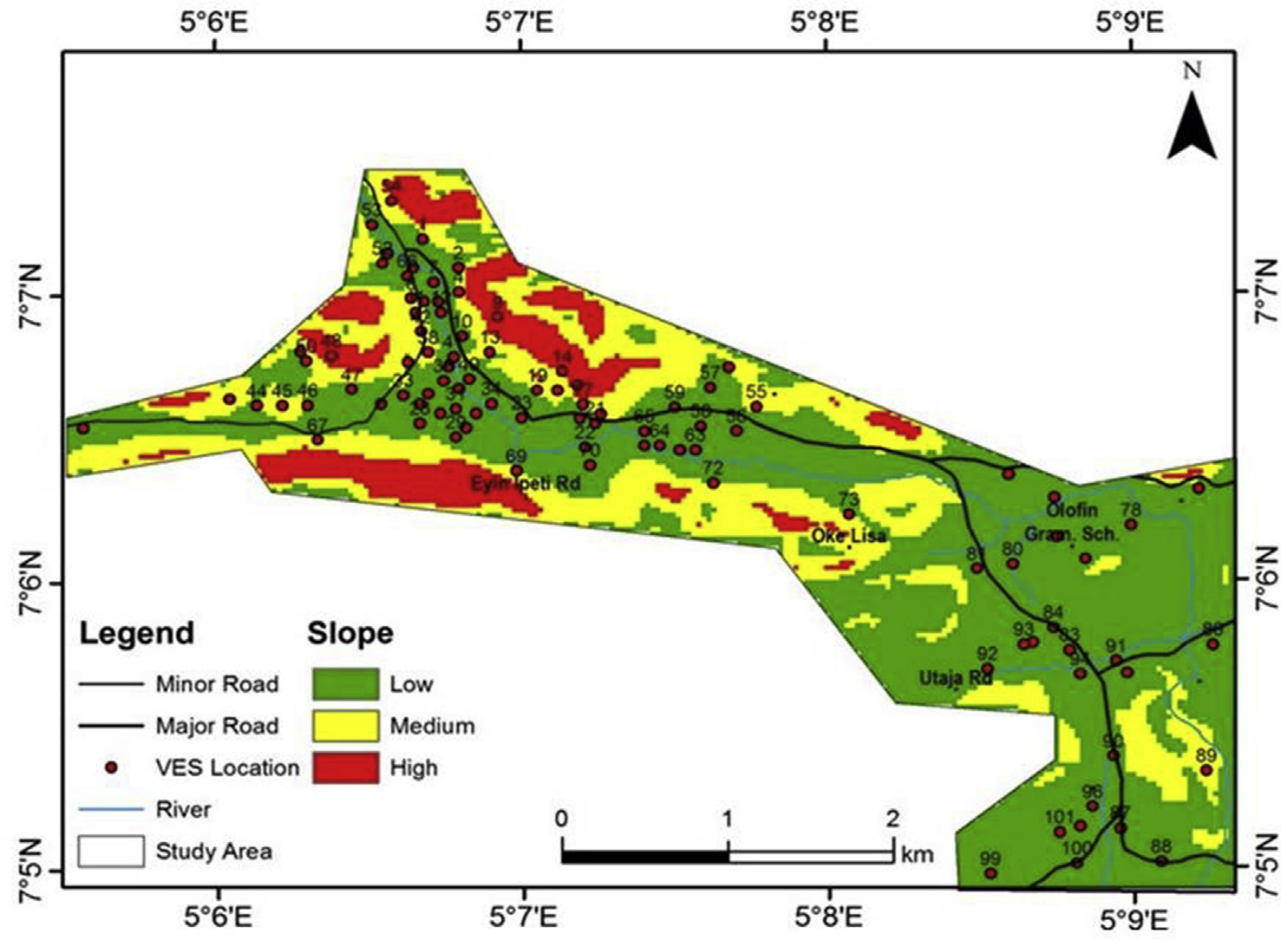
Slope map of the study area.

The slope map of the study area is as shown in Figure 8. Slope is an important factor in groundwater potential zoning. The higher the degree of slope, the more rapid the run-off will be. This increases the erosion rate with poor recharge potential (Hammouri et al., 2012). The study area was grouped into three slope classes; 0^o^ – 9.48°, 9.49–22.52° and 22.53–50.36° indicating low, medium, and high degree of slope respectively. Areas with low degree of slope (in green colour) can be considered to be hydrogeologically significant as they are characterized by nearly flat terrain with high infiltration rate.

#### 4.1.4 Aquifer resistivity

**Figure 9:**
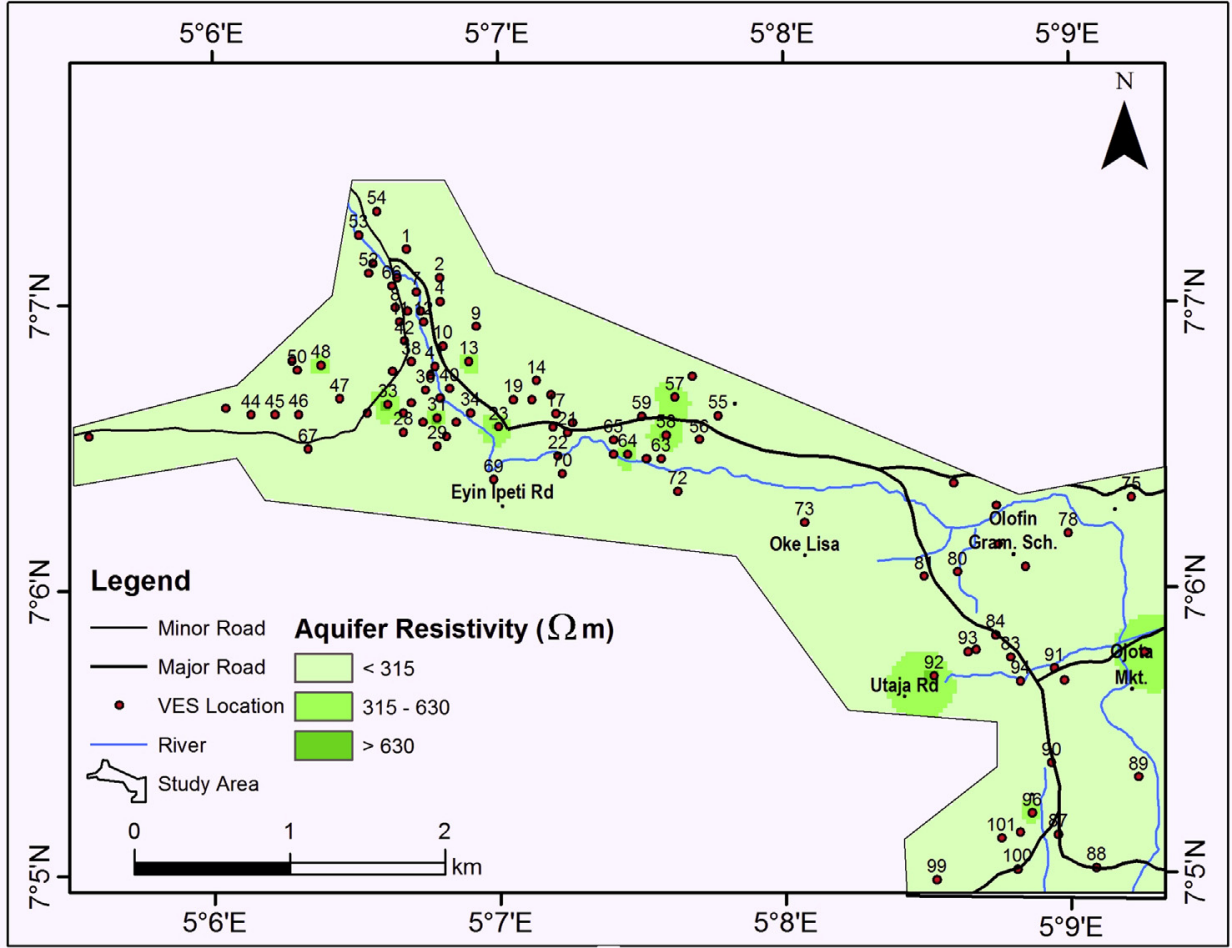
Aquifer resistivity map of the study area.

**Figure 10:**
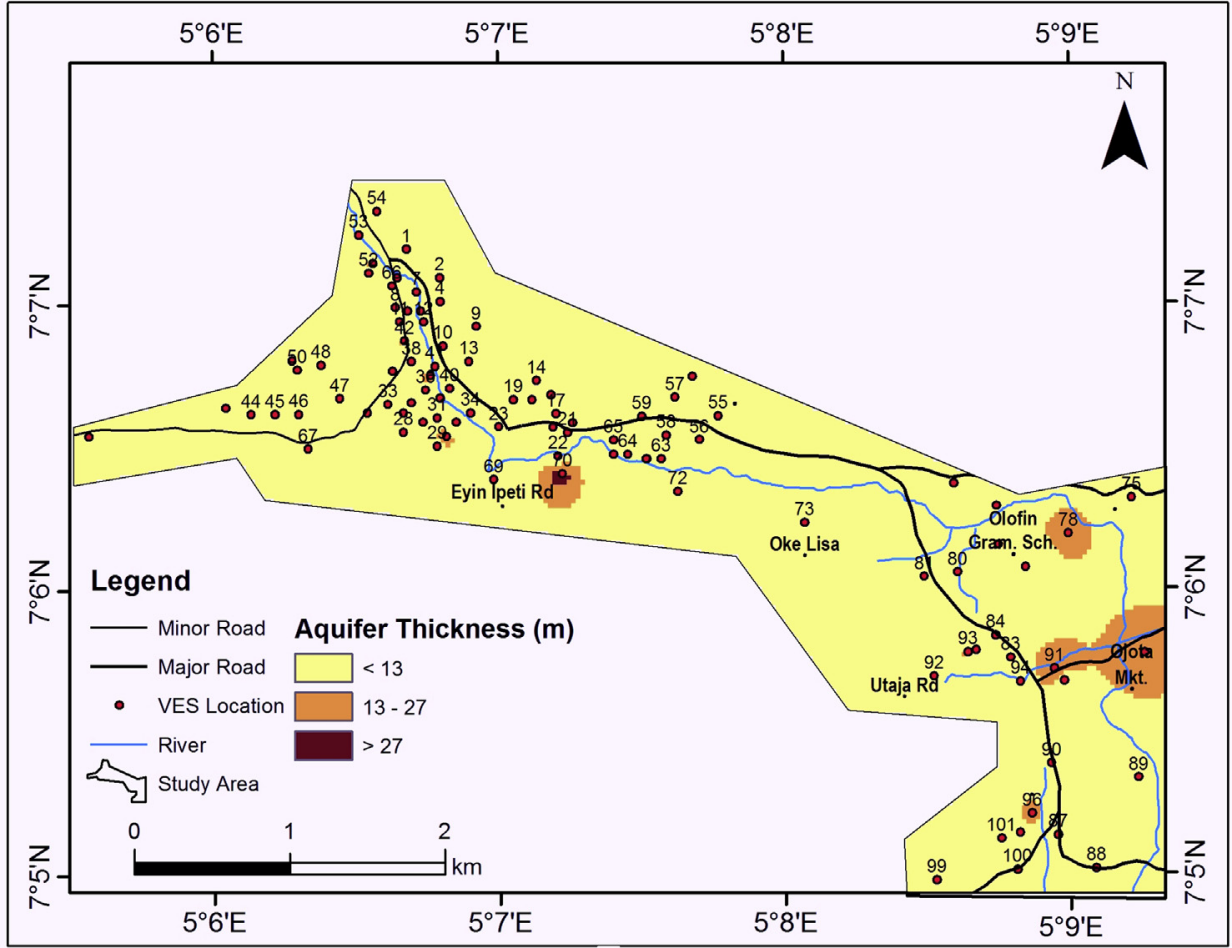
Aquifer thickness map of the study area.

The aquifer resistivity map as shown in Figure 9 reveals that the study area is characterized by aquifer unit resistivity ranging from 0 – 891 Ωm but generally less than 150 Ωm (Table 1) thereby indicating a composition of clay/sandy clay. The thickness of the aquifer ranges from 0 – 36.6 m but generally less than 13 m (Figure 10). They are isolated thick aquifer unit (>27 m) within the study area as shown in Figure 10. The aquifer unit is predominantly clayey material and relatively thin (˃13 m). This is an indication that, the aquifer unit is less permeable with tendency for low yield.

#### 4.1.5 Hydraulic conductivity

**Figure 11:**
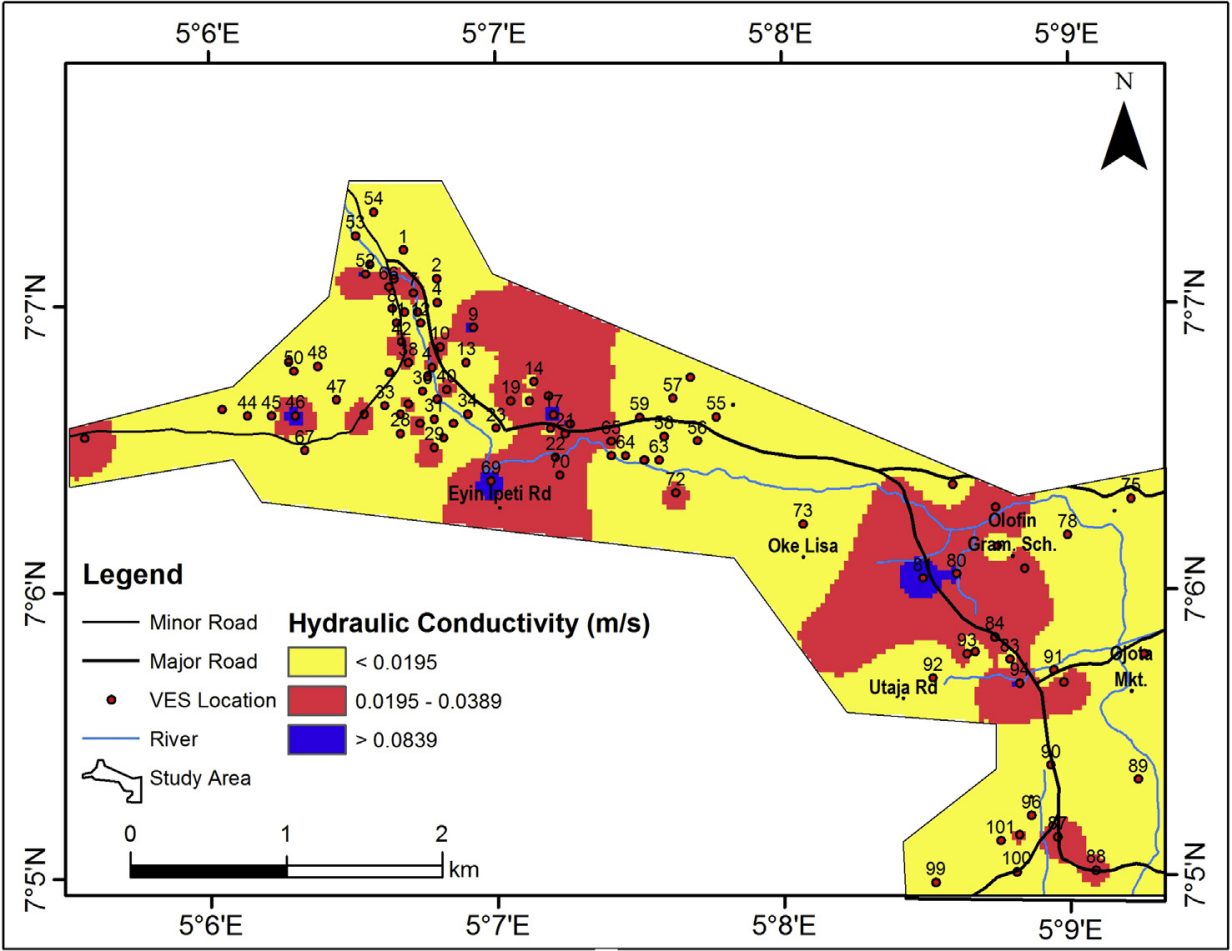
Aquifer unit hydraulic conductivity map of the study area.

Hydraulic conductivity is a measure of the ease with which a fluid will pass through a medium. Figure 11 shows the hydraulic conductivity (K) distribution in the study area. The aquifer hydraulic conductivity (K) ranges from 0 – 0.0519 m/s (Table 2) but generally less than 0.0195 m/s (Figure 11). The high range of hydraulic conductivity of the aquifer may be due to the heterogeneity nature of the aquifer, a condition responsible for wide range in hydraulic conductivity (George et al., 2015). The area is predominantly of low hydraulic conductivity (<0.0195 m/s), indicating that groundwater flow in the area is not simple but complex because of the geologic control of the confined aquifer.

#### 4.1.6 Transmissivity

**Figure 12:**
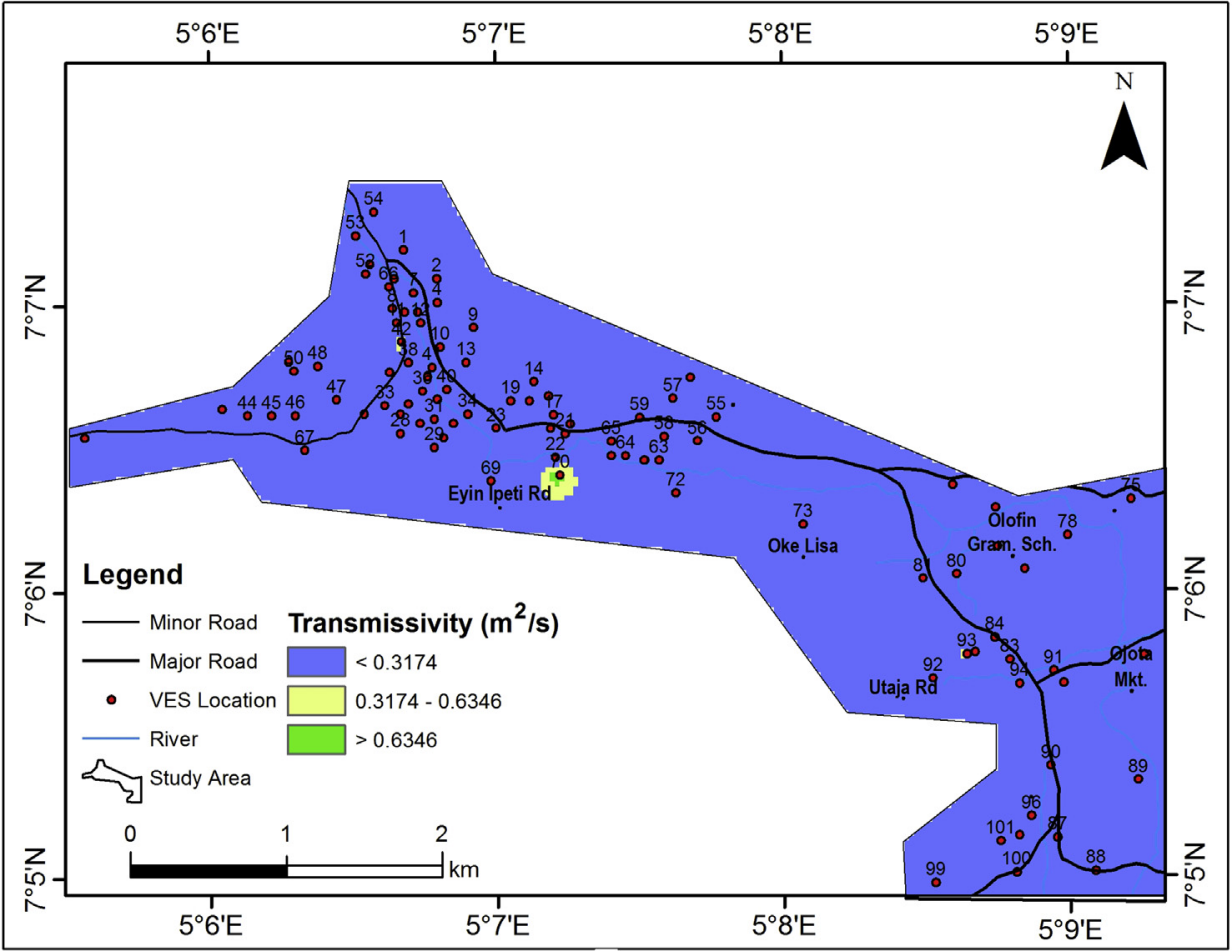
Aquifer unit transmissivity map of the study area.

The transmissivity (T) value ranges from 0 – 0.8739 m^2^/day (Table 2), but generally less than 0.3174 m^2^/day (Figure 12). Area with high transmissivity values can be identified as area of high water bearing potential and Aquifer materials are known to be relatively permeable to fluid movement. The average transmissivity value of the study area is 0.0643 m^2^/day. Thus, indicating that the area is of low groundwater potential.

#### 4.1.7 Coefficient of anisotropy map of the area

**Figure 13:**
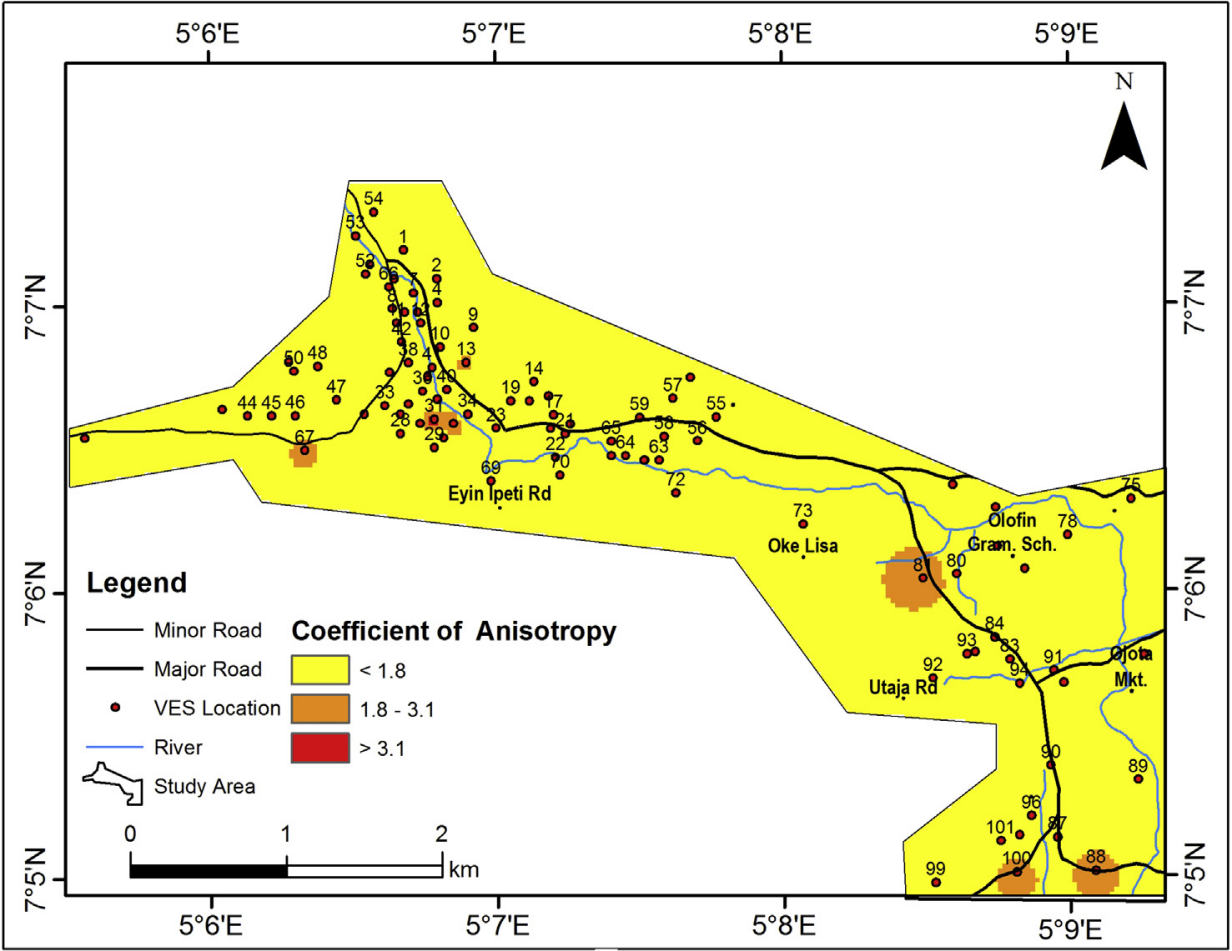
Coefficient of anisotropy map of the study area.

Figure 13 shows the coefficient of anisotropy map of the study area. The study area is characterized by coefficient of anisotropy (λ) ranging from 0.6456 – 4.0349 Ω (Table 2). The determination of the coefficient of anisotropy of the area becomes necessary to ascertain the development of secondary porosity within rocks in the area, since there are indications of fracture/partly weathered layer aquifer units from the interpretation of the geologic sections.

Coefficient of anisotropy (λ) is a measure of heterogeneity, which may results from fracturing, discontinuities or presence of clay (Olayanju, 2003). Fresh Basement rock usually exhibit anisotropy index of ʽ 1 ʼ and this value increases as the Basement rock is exposed to weathering and fracturing. Accordingly, few isolated closures of high coefficient of anisotropy (>1.8725) have been identified in the northwestern and southeastern parts of the study area where it was suspected to be underlain by Basement fracture. This area can be considered to be hydrogeologically significant. The identified areas of high coefficient of anisotropy correlate with areas with relatively high lineament density. VES stations indicate the presence of partly weathered/fractured Basement.

### 4.2 Groundwater potential map of the study area

**Figure 14:**
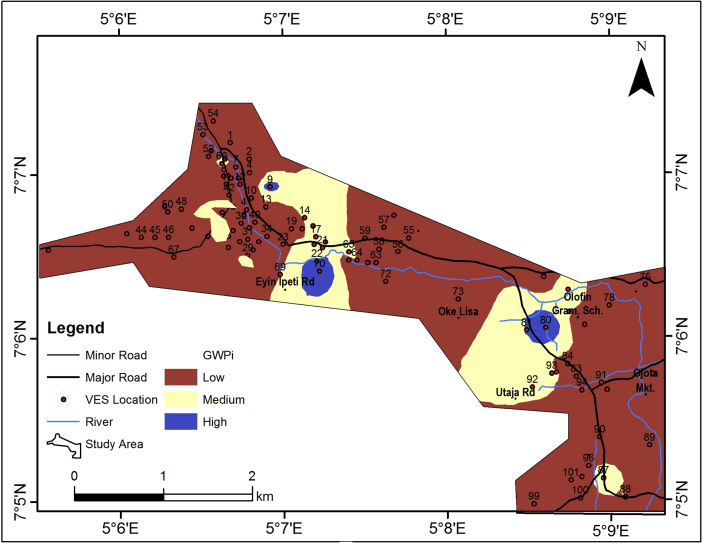
Groundwater potential map of the study area.

The groundwater potential index (GWPI) values for the study area vary between 50 and 250 (Table 2). This was used to generate the groundwater potential model of the area. The groundwater potential model, upon consideration of the aquifer resistivity, aquifer thickness, coefficient of anisotropy, hydraulic conductivity and transmissivity of the aquifer unit, the slope, drainage density and the lineament density is as shown in Figure 14. The groundwater potential model has categorized the study area into three classes which include high, medium and low groundwater potential classes. The major parts of the study area falls within the low groundwater potential zones (about 85%). Therefore, the groundwater potential of the study area can be considered to be of low level rating.

### 4.3 Vulnerability assessment of the aquifer units in the study area

The assessment of the vulnerability of the aquifer units to contaminants has been undertaken by investigating the vulnerability capacity of the layer overlying the aquifer units in the area (Vadose zone) to offer protection to the underlying aquifer units. Hence, the thickness and the longitudinal conductance of the vadoze zone are taken into consideration.

**Figure 15:**
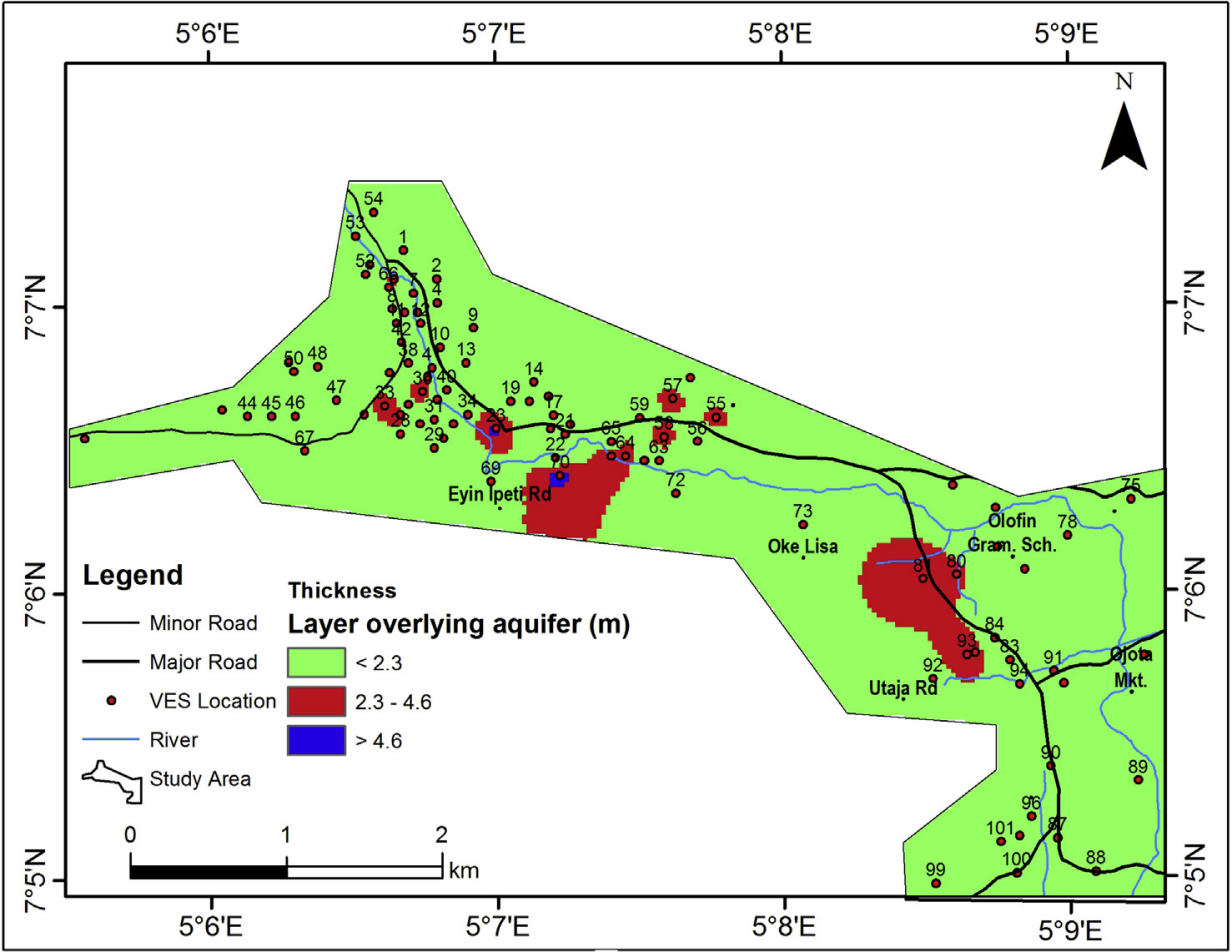
Thickness map of the layer overlying the aquifer (vadose zone).

The vulnerability of the aquifers in the study area to contamination is estimated by considering the longitudinal conductance and the thickness of the geo-electric layers overlying the aquifers. These zones are often referred to as the Vadoze zone. Also, the lineament density and hydraulic conductance earlier considered for groundwater accumulation also play significant role in aquifer vulnerability mapping. The thickness of the vadose zone ranges between 0 and 5.3 m (Table 2), but generally less than 2.3 m (Figure 15). Given the general thin nature of the vadose zone, the resident time of potential contaminants from the surface into the vadoze zone will be short and the underlying aquifer units can easily be impacted.

**Figure 16:**
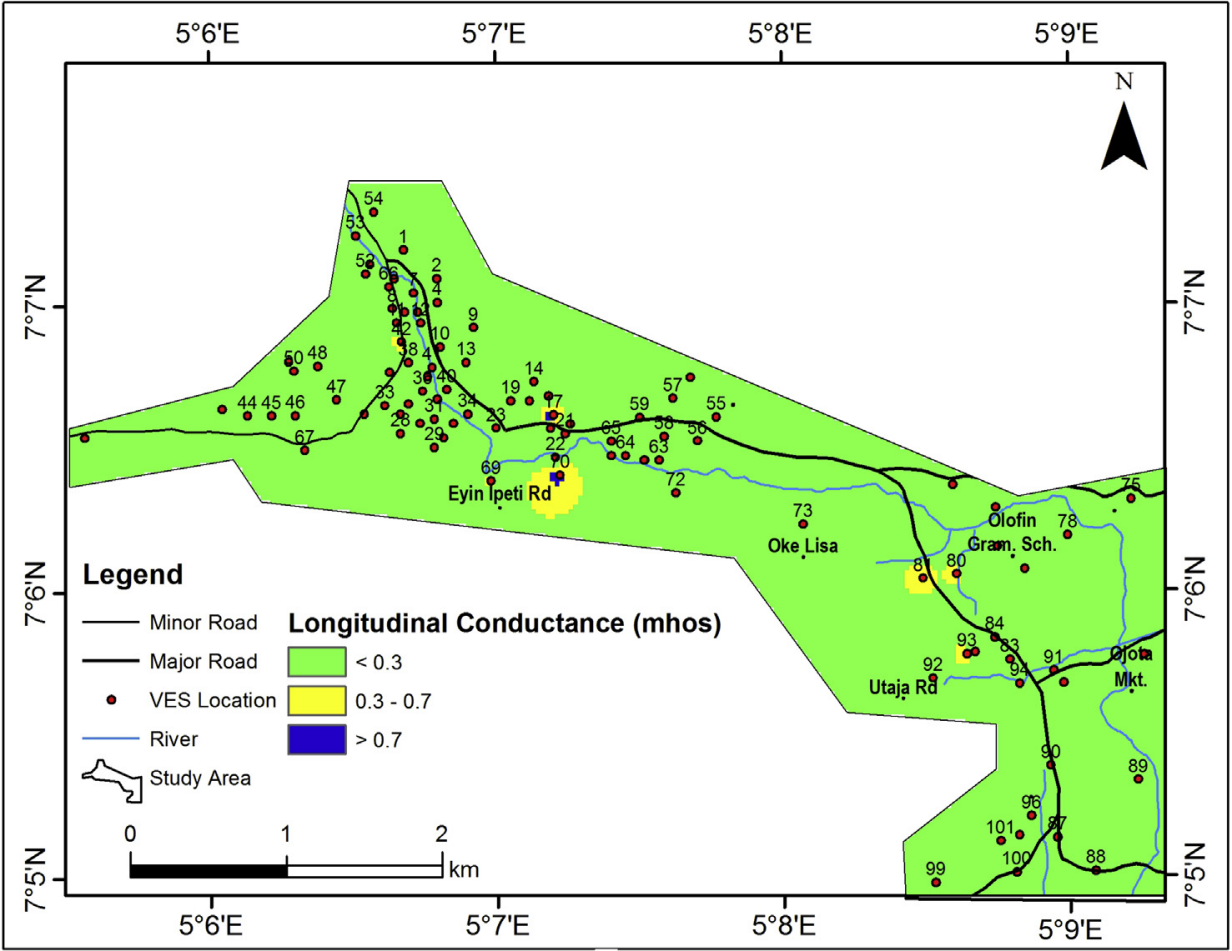
Longitudinal conductance map of the study area.

**Figure 17:**
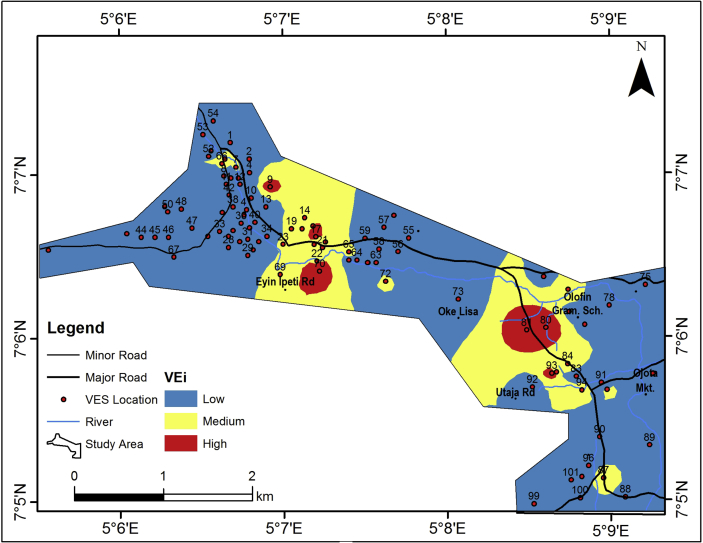
Aquifer vulnerability map of the study area.

The longitudinal conductance of the vadoze zone which also provides a measure of the aquifer protective capacity (APC) is presented in Figure 16 and the value ranges from 0.0038 – 0.9448 mhos across the area. Highly impervious materials such as clay and shale usually have high longitudinal conductance values (resulting from their low resistivity values) while pervious materials such as sand and gravels have low longitudinal conductance values (resulting from their high resistivity values). Thus, high longitudinal conductance value indicates a good protective capacity while low longitudinal conductance values are associated with poor/weak protective capacity (Oladapo and Akintorinwa, 2007; Abiola et al., 2009; Akintorinwa and Olowolafe, 2013). Using Table 3, major parts of the study area offers weak to medium protection for the underlying aquifers based on their characteristic low longitudinal conductance (˂ 1). The vulnerability model, upon consideration of the thickness and longitudinal conductance of the vadose zone, the hydraulic conductivity of the aquifer units, the slope and the lineament density is presented in Figure 17. The Vulnerability Index (VI) values for the study area very between 60 and 300 (Table 2). The aquifer vulnerability model has categorized the study area into three classes which include high, medium and low vulnerability classes.

**Table 3:** Longitudinal conductance/protective capacity rating (modified after Oladapo and Akintorinwa, 2007).

Longitudinal Conductance (mhos)	Protective Capacity Rating
>10	Very Good
1–10	Good
0.05–1	Medium
<0.05	Weak

The major parts of the study area falls within the low vulnerable zones (about 80%). The medium to high vulnerable zones account for about 20%. Therefore, the underlying aquifers in the study area can considered to be relatively protected from potential infiltration of contaminants from the surface.

### 4.4 Validation of the groundwater potential model

The depth of the hand-dug wells selected for validation of results ranges from 4.1 to 11.7 m. The static water level ranges from 2.1 to 9.5 m and the water column varies from 1.1 to 6.4 m. The interviewed hand-dug well owners, remarks were made as stated in Table 4.

**Table 4:** Hand-dug wells parameters.

S/N	Coordinate		Well Depth (m)	Static Water Level (m)	Water Colum (m)	Rating Based on Groundwater Potential Map	Remark
	Easting (Em)	Nothing (Nm)					
1	737036	784926	6.8	5.1	1.7	L	∗∗∗
2	736903	784839	8	6.5	1.5	L	∗∗∗
3	736694	785367	11.7	5.8	5.9	H	∗
4	736917	785413	10.5	6.5	4	M	∗
5	737308	784595	5.6	3.9	1.7	L	∗∗∗
6	737178	784566	7.2	5.2	2	L	∗∗
7	737015	784577	7.8	6	1.8	L	∗∗∗
8	737408	783626	6.5	3	3.5	M	∗
9	737253	783521	8.4	4.2	4.2	M	∗
10	735207	783551	6.5	4	2.5	L	∗∗
11	737002	783538	7.5	5.3	2.2	L	∗∗
12	737413	784078	6.1	3.8	2.3	L	∗∗
13	737468	784092	6.5	4.5	2	L	∗∗
14	735014	786549	8.6	6.8	1.8	L	∗∗∗
15	735143	786552	5.7	4	1.7	L	∗∗∗
16	735139	786568	6.8	4.3	2.5	L	∗∗∗
17	735044	786304	6.7	5.2	1.5	L	∗∗∗
18	735022	786189	6.4	4.9	1.5	L	∗∗∗
19	734768	786267	10	8.3	1.7	L	∗∗∗
20	734724	786098	7	4.9	2.1	L	∗∗
21	734705	786332	8.7	6.7	2	L	∗∗
22	734744	786452	11.5	9.5	2	L	∗∗
23	734419	786180	6.4	2.5	3.9	M	∗
24	734402	786011	9.2	5.1	4.1	M	∗
25	734089	782303	6.4	4.9	1.5	L	∗∗∗
26	734033	786128	10.3	3.9	6.4	H	∗
27	734219	785914	5.6	2.2	3.4	M	∗
28	733499	786679	5.1	Dry	0	L	∗∗∗∗
29	733473	786744	5.6	3.5	2.1	L	∗∗
30	732839	786210	4.3	2.8	1.5	L	∗∗∗
31	733125	787396	5.3	3.4	1.9	L	∗∗∗
32	737544	783890	9.5	5.5	4	M	∗
33	737632	783794	10.2	6	4.2	M	∗
34	737879	783582	6.2	Dry	0	L	∗∗∗∗
35	737530	783572	11.5	7.3	4.2	M	∗
36	737350	784789	7.3	Dry	0	L	∗∗∗∗
37	737687	784891	4.1	2.1	2	L	∗∗
38	736782	786091	8.5	6.5	2	L	∗∗
39	737154	786103	7.8	6.3	1.5	L	∗∗∗
40	737969	786009	6.9	5.8	1.1	L	∗∗∗
41	735444	785891	8.2	6.4	1.8	L	∗∗∗
42	735208	785992	11.2	8.8	2.4	L	∗∗
43	734835	786034	9.2	7.1	2.1	L	∗∗
44	733275	786250	6.4	4.2	2.2	L	∗∗
45	732788	786424	5.4	3.3	2.1	L	∗∗
46	732437	786339	5.4	Dry	0	L	∗∗∗∗
47	731862	786349	4.9	2.8	2.1	L	∗∗
48	731399	786413	7.8	6.5	1.3	L	∗∗∗

∗Productive throughout the year.

∗∗Reduced in productive during dry season.

∗∗∗Dry up during dry season.

∗∗∗∗Dry up at time of measurement.

**Figure 18:**
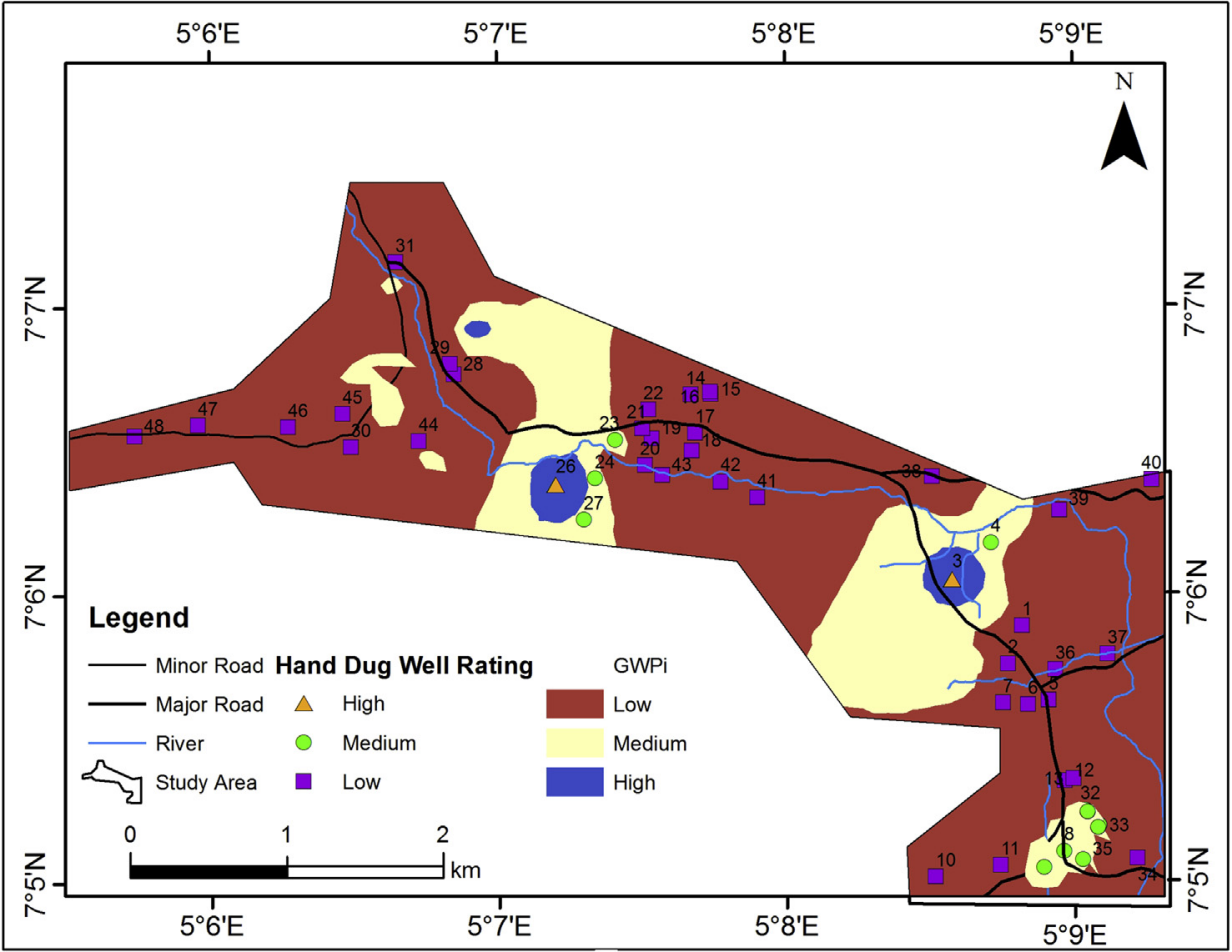
Hand-dug well location on the groundwater potential model.

The hand-dug well locations were superimposed on the established groundwater potential model (Figure 18). The hand-dug wells with relatively large water volume (3.9–6.4 m) fall within the medium/high groundwater potential zones; while the wells with relatively small water volume (1.1–2.5 m) fall within low groundwater potential zone (Table 4 and Figure 18). The hand-dug well that falls within the medium/high groundwater potential zones are productive throughout the year and those that falls within the low groundwater potential were dry up at the time of taking static water level or dry up during dry season (October–April) (Table 4).

Out of forty-eight (48) hand-dug wells used for the validation, 77% falls within the low groundwater potential zones and they are the wells with low water volume, which dry up during the dry season. The remaining 23% that are productive throughout the year falls within medium/high groundwater potential zones. This shows that, the established groundwater potential model in this research validate the groundwater productivity of the study area. Hence, the produced groundwater potential model has good prediction accuracy.

## 5 Conclusions

In this research, the electrical resistivity, remote sensing and geographic information system (GIS) were used to evaluate geoelectro-hydrualic parameters of a typical Basement Complex in terms of its groundwater potentiality and vulnerability to contamination. Three to four geo-electric layers namely top soil, weathered layer, fractured basement and fresh basement were delineated in the study area. The weathered and fractured basement layers constitute the aquifer units through which groundwater can be tapped. The aquifer units are characterized by clay/clayey sand. The clay unit due to its porosity and low permeability will exhibit low groundwater potential unit. The hydrogeological characteristics of the clayey sand units suggest moderate groundwater potentiality due to its higher porosity and permeability in comparison to the clay aquifer unit.

The first and second order information obtained from the geo-electric parameters and remote sensing data namely longitudinal conductance, coefficient of anisotropy, aquifer thicknesses and resistivity, thickness of layer overlying aquifer unit, hydraulic conductivity, transmissivity, slope, drainage and lineament density assisted in the development of the groundwater potential and vunerability maps of the study area.

Using the application of Arc GIS software, the relevant hydrogeological thematic maps based on these parameters were produced. Applying the inverse distance weighting (IDW) in the context of analytical hierarchy process (AHP) data mining approach, the rated and weighted thematic layers were integrated in GIS environment to compute groundwater potential index (GWPI) and aquifer vulnerability index (VI) for the area. The computed GWPI ranges from 50 to 200 while the computed VI ranges from 50.1 to 250.5. The area was classified into three groundwater potential and aquifer vulnerable zones (low, medium and high).

About 80% of the area falls within the low groundwater potential rating, while the rest falls within the medium/high groundwater potential rating. Hence, the area can be generally rated to be of low groundwater potential. The major parts of the area also falls within the low vulnerable zone (about 80%), while the medium/high vulnerable zones account for about 20%. Therefore, the underlying aquifer in the area is relatively protected from the infiltration of surface contaminants.

The medium and high groundwater potential zones predicted in the area correlates with the hand-dug wells that are productive throughout the year. The predicted low groundwater potential correlates hand-dug wells that are non-productive or dry up during the dry season. Hence, the predicted groundwater potential model is of good accuracy.

## Declarations

### Author contribution statement

O. J. Akintorinwa, M. O. Atitebi: Conceived and designed the experiments; Performed the experiments; Analyzed and interpreted the data; Contributed reagents, materials, analysis tools or data; Wrote the paper.

A. Akinlalu: Analyzed and interpreted the data; Contributed reagents, materials, analysis tools or data; Wrote the paper.

### Funding statement

This research did not receive any specific grant from funding agencies in the public, commercial, or not-for-profit sectors.

### Competing interest statement

The authors declare no conflict of interest.

### Additional information

No additional information is available for this paper.

## References

[bib1] Abiola O., Enikanselu P.A., Oladapo M.I. (2009). Groundwater potential and aquifer protective capacity of overburden units in ado-ekiti. Int. J. Phys. Sci..

[bib2] Adeeko T.O., Samson D.O., Umar M. (2019). Geophysical survey of basement ComplexTerrain using electrical resistivity method for groundwater potential. World News Nat. Sci..

[bib3] Adeyeye O.A., Ikpokonte E.A., Arabi S.A. (2019). GIS-based groundwater potential mapping within Dengi area, North Central Nigeria. Egyp. J. Rem. Sens. Space Sci..

[bib4] Adiat K.A.N., Nawawi M.N.M., Abdullah K. (2012). Assessing the accuracy of GIS-based elementary multi criteria decision analysis as a spatial prediction tool-A case of predicting potential zones of sustainable groundwater resources. J*. Hydrol.*.

[bib5] Afolayan J.F., Olorunfemi M.O., Afolabi O. (2004). Geoelectric/electromagnetic VLF survey for groundwater development in a basement terrain – a case study. IFE J. Sci..

[bib6] Akinlalu A.A., Adegbuyiro A., Adiat K.A.N., Akeredolu B.E., Lateef W.Y. (2017). Application of multi-criteria decision analysis in prediction of groundwater resources potential: a case of oke-ana, Ilesa area, southwestern, Nigeria. NRIAG J. Astron. Geophys..

[bib7] Akintorinwa O.J., Olowolafe T.S. (2013). Geoelectric evaluation of groundwater prospect within zion estate, akure, southwest, Nigeria. Int. J. Water Resour. Environ. Eng..

[bib8] Ako B.D., Olorunfemi M.O. (1989). Geoelectric survey for groundwater in the newer Basalts of Vom plateau state. Nig J. Min. Geol..

[bib9] Al-Abadi A.M., Al-Shamma’a A.M., Aljabbari M.H. (2017). A GIS-based DRASTIC model for assessing intrinsic groundwater vulnerability in northeastern Missan governorate, southern Iraq. Appl. Water Sci..

[bib11] Alile O.M., Ujuambi, Evbuomwan I.A. (2011). Geoelectric investigation of groundwater in obaretin-Iyanorno locality, edo state, Nigeria. J. Geol. Min. Res..

[bib12] Anifowose A.Y.B., Kolawole F. (2012). Emplacement tectonics of the Idanre, batholith, west africa. Comunicações Geológicas.

[bib13] Aniya F.B., Shoeneick K. (1992). Hydrogeological investigation of the aquifer of Bauchi area. J. Min. Geol..

[bib14] ArcGIS (2010). [GIS Software]. Version 10.0.

[bib15] Arsène Meying., Bidichael Wahile, Wassouo Elvis, Gouet Daniel., Ndougsa-Mbarga Théophile., Kuiate Kelian., Jean Daniel Ngoh (2018). Hydrogeophysical investigation for groundwater resources from electrical resistivity tomography and self-potential data in the Méiganga area, adamawa, Cameroon. Int. J.Geophys..

[bib16] Babiker I.S., Mohammed M.A.A., Hiyama T., Kato K. (2005). A GIS-based DRASTIC model for assessing aquifer vulnerability in Kakamigahara Heights, Gifu Prefecture, Central Japan. Sci. Total Environ..

[bib17] Bayode S., Ojo J.S., Olorunfemi M.O. (2006). Geoelectric characterization of aquifer types in the basement complex terrain of parts of osun state, Nigeria. Global J. Pure Appl. Sci..

[bib18] Colins J., Sashikkumar M.C., Anas P.A., Kirubakaran M. (2016). GIS-based assessment of aquifer vulnerability using DRASTIC Model: a case study on Kodaganar basin. Earth Sci. Res. J..

[bib19] ERDAS (2001). Erdas IMAGINE Tour Guides.

[bib20] ESRI (2001). Linear Referencing and Dynamic Segmentation in ArcGIS 10.1. Redlands, CA.

[bib21] Evans U.F., George N.J., Akpan A.E., Obot I.B., Ibot A.N. (2010). A study of superficial sediments and aquifers in parts of Uyo local government area, AkwaIbom State, Southern Nigeria, using electrical sounding method. Eur. J. Chem..

[bib22] Foster S., Hirata R., Gomes D., Elia M.D., Paris M. (2002). Groundwater Quality Protection A Guide for Water Utilities, Municipal Authorities and Environment Agencies.

[bib23] George N.J., Akpan A.E., Obot I.B. (2010). Resistivity study of shallow aquifer in parts of southern Ukanafun Local government area, Akwa-Ibom State. Eur. J. Chem..

[bib24] George N.J., Emah J.B., Ekong U.N. (2015). Geophysical properties of hydrogeological units in parts of Niger Delta, southern Nigeria. J. Afr. Earth Sci..

[bib25] Leica Geosystems (1999). Earth Resources Data Analysis System (ERDAS) Field Guide.

[bib26] Hammouri N., El-Naqa A., Barakat M. (2012). An integrated approach to groundwater exploration using remote sensing and geographic information system. J. Water Resour. Protect..

[bib27] Helaly A.S. (2017). Assessment of groundwater potentiality using geophysical techniques in Wadi Allaqi basin, Eastern Desert, Egypt – case study. NRIAG J. Astron. Geophys..

[bib28] Ibuot J.C., Akpabio G.T., George N.J. (2013). A survey of repository of groundwater potential and distribution using geoelectrical resistivity method in Itu L.G.A., AkwaIbom State, Southern Nigeria. Cent. Eur. J. Geosci..

[bib29] ITC (2001). Academic User’s Guide.

[bib30] Jamrah A., Al-Futaisi A., Rajmohan N., Saif Al-Yaroubi (2008). Assessment of groundwater vulnerability in the coastal region of Oman using DRASTIC index method in GIS environment. Environ. Monit. Assess..

[bib31] Jha M.K., Chowdhury A., Chowdary V.M., Peiffer S. (2007). Groundwater management and development by integrated remote sensing and geographic information systems: prospects and constraints. Water Resour. Manag..

[bib32] Khodadadi N., Asadollahfardi G., Heidarzadeh N. (2015). Application of a GIS-based Drastic model and groundwater quality index method for evaluation of groundwater vulnerability A case study Sefid-Dasht. Water Supply.

[bib33] Khodadbakhshi N., Heidarzadeh N., Asadollahfardi G. (2017). Vulnerability assessment of an aquifer using modified GIS-based methods. Am. Water Works Assoc. J..

[bib34] Magesh N.S., Chandrasekar N., Soundranayagam J.P. (2012). Delineation of groundwater potential zones in Theni district, Tamil Nadu, using remote sensing, GIS and MIF techniques. Geosci. Front..

[bib35] Mogaji K.A., Lim H.S., Abdullah K. (2014). Regional prediction of groundwater potential mapping in a multifaceted geology terrain using GIS-based Dempster–Shafer model. Arab. J. Geosci..

[bib36] Ocan T. (1991). Petrogenesis of the Rock Units of Idanre, Southwestern Nigeria.

[bib37] Odong P.O. (2013). Groundwater potential evaluation and aquifer characterization using resistivity method in Southern Obubra, Southeastern Nigeria. Int. J. Environ. Sci..

[bib38] Offodile M.E. (2002). Groundwater Study and Development in Nigeria.

[bib39] Oladapo M.I., Akintorinwa O.J. (2007). Hydrogeophysical study of ogbese south western Nigeria. Global J. Pure Appl. Sci..

[bib40] Oladapo M.I., Mohammed M.Z., Adeoye O.O., Adetola B.A. (2004). Geo-electrical investigation of Ondo state housing coperation estate, Ijapo, akure, south western Nigeria. J. Min. Geol..

[bib41] Oladunjoye M.A., Korode I.A., Adefehinti A. (2019). Geoelectrical exploration for groundwater in crystalline basement rocks of Gbongudu community, Ibadan, southwestern Nigeria. Global J. Geol. Sci..

[bib42] Olayanju G.M. (2003). Delineation of fault assisted aquifer using tripotential wenner array-technique around Ita-oniyan industrial layout, akure, Nigeria. Niger. J. Pure Appl. Phys. (NJPAP).

[bib43] Olorunfemi M.O., Fasuyi S.A. (1993). Aquifer types and the geo-electric/hydrogeologic characteristics of part of the central basement terrain of Nigeria (Niger state). J. Afr. Earth Sci..

[bib44] Omosuyi G.O. (2010). Geoelectric assessment of groundwater prospect and vulnerability of overburden aquifers at Idanre, southwestern Nigeria. Ozean J. App. Sci..

[bib45] Owoyemi F.B. (1996). A Geological-Geophysical Investigation of Rain-Induced Erosional Features in Akure metropolis.

[bib46] Kamlesh Prasad, Shukla J.P. (2014). Assessment of groundwater vulnerability using GIS-based Drastic technology for the basaltic aquifer of Burhner watershed, Mohgaon block, Mandla (Indian). Curr. Sci..

[bib47] Rahaman M.A. (1988). Recent Advances in the Study of the Basement Complex of Nigeria. Precambrian Geology of Nigeria.

[bib48] Shahab A., Shihua Q., Rad S., Keita S., Khan M., Adnan S. (2019). Groundwater vulnerability assessment using GIS-based DRASTIC method in the irrigated and coastal region of Sindh province, Pakistan. Nord. Hydrol.

[bib49] Shankar Babu Pokharel (2007). Remote Sensing and GIS Analysis of Spatial Distribution of Fracture Pattern in the MakranAccretionary Prism, Southeast Iran.

[bib50] Srinivasan K., Poongothai S., Chidambaram S. (2013). Identification of groundwater potential zone by using GIS and electrical resistivity techniques in and around the Wellington reservoir, Cuddalore district, Tamilnadu, India. Eur. Sci. J. ESJ.

[bib52] Vander Velpen B.P.A. (2004). Resist Version 1.0. M.Sc. Research Project.

[bib53] Venkateswaran S., Vijay M. Prabhu, Karuppannan S. (2014). Delineation of groundwater potential zones using geophysical and GIS techniques in the sarabanga sub basin, cauvery river, Tamil nadu, India. Int. J. Curr. Res. Acad. Rev..

[bib54] Zeyad J.A. (2013). Lineament extraction for assessment of groundwater potential in west of Iraq. Euphrates J. Agric. Sci..

[bib55] Zghibi A., Merzougui A., Chenini I., Ergaieg K., Zouhri L., Tarhouni J. (2016). Groundwater vulnerability analysis of Tunisian coastal aquifer: an application of DRASTIC index method in GIS environment. Groundwater Sustain. Develop..

